# Supplementary material for: The non-linear impact of data handling on network diffusion models
Source: Patterns (N Y). 2021 Nov 26;2(12):100397. doi: 10.1016/j.patter.2021.100397 (PMC8672192; doi:10.1016/j.patter.2021.100397)
Supplement: Document S1. Supplemental experimental procedures and Figures S1–S8 [file mmc1.pdf]

Patterns, Volume *2*

Supplemental information

The non-linear impact of data handling on network diffusion models

James Nevin, Michael Lees, and Paul Groth

Supplemental Experimental Procedures

1 NETWORK PROPERTY CHANGES

Supplementary Figure 1 shows the impact of node removal on various network properties for the networks tested. The impact is most visible in the closeness centralities, where we see a non-linear impact of node removal. The average clustering coefficient is robust to node removal. The normalised average degree does show some change (especially in the BA network), but these changes are of relatively low magnitude (0.1 to 0.097). These same data handling errors showed non-linear behaviour on diffusion model output, highlighting that data handling's impact on network properties does not necessarily mirror its impact on diffusion models.

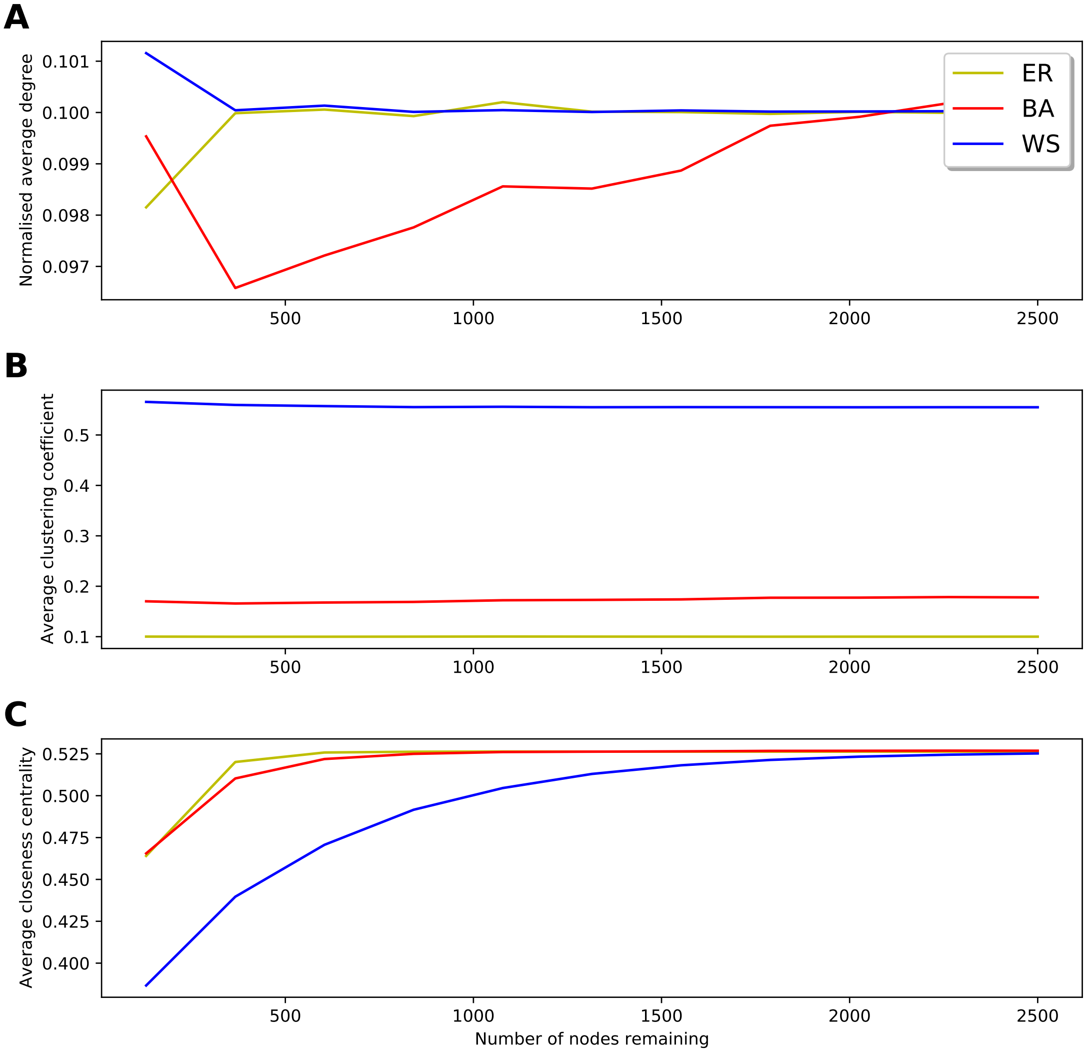

Supplementary Figure 1. Network property changes as nodes are removed. Network normalised average degree (A), average clustering coefficient (B), and average closeness centrality (C) for various nodes remaining

2 EDGE REMOVAL

For each of the 20 initial networks of each topology, we run the model 10 times after each batch of random edge removal. In the SIR case, edge removal is continued until the normalised average degree has been reduced to approximately 0.05 (roughly 50% of edges removed) prior to running multiple iterations of the

diffusion model, as all trials result in full spread of the infection at normalised average degrees above this. The results of random edge removal are generally very similar to those seen in random node removal when using high-level metrics.

2.1 SIR Model

Supplementary Figure 2 replicates Figure 6 for edge removal, showing the proportion of the population becoming infected when running the SIR model (and its change) as we vary the normalised average degree of the networks. As we remove edges and thus lower the average degree of the network, there is a reduction in the spread of the infection. The rate at which this change in spread happens is almost identical between the ER and WS networks, but differs in the BA network; in the BA network, the spread starts to reduce earlier as one removes edges, but does not change as rapidly as in the ER and WS networks. Additionally, this change in spread is not linear, beginning more slowly as one removes edges and gradually increasing. This can be seen in the change plot (this change has been scaled so that it represents the change relative to a change of 0.001 in the normalised average degree). We can see that this change is highly non-linear, with peaks observed at an average degree of around 0.01. Again, we note that this differs between the BA network and the ER and WS networks; the BA change graph has a lower peak with a less steep gradient.

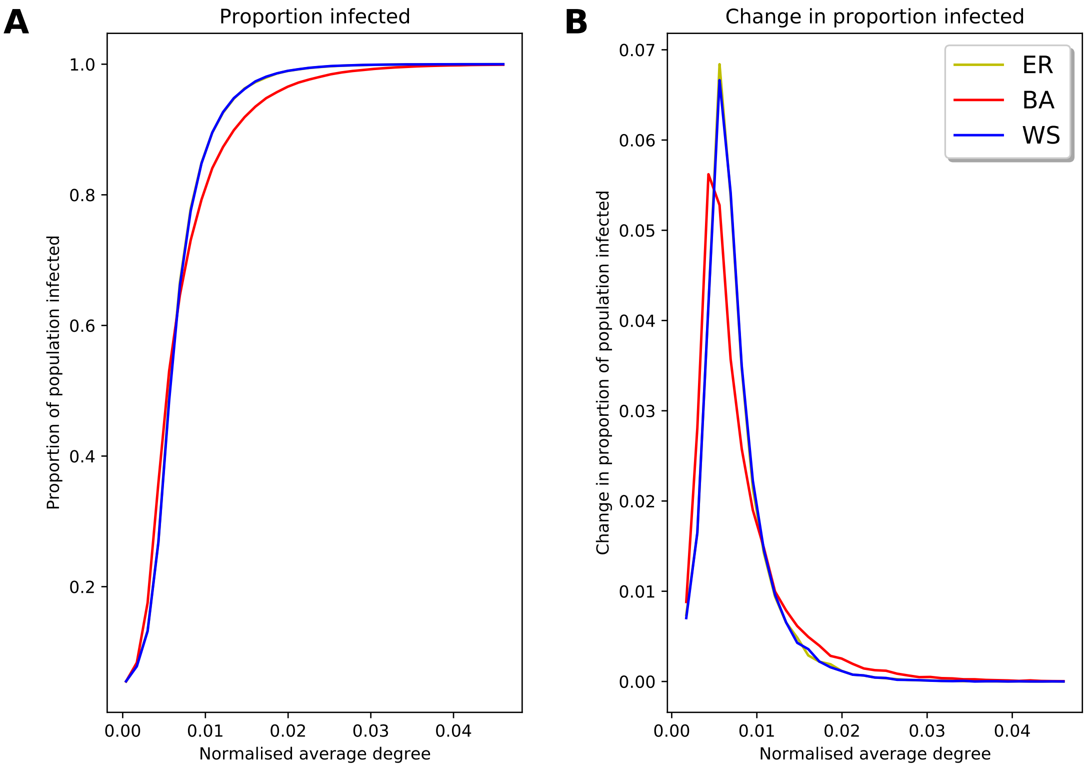

Supplementary Figure 2. Total infection for SIR model on synthetic networks with random edge removal. Proportion of population becoming infected for varying average node degree (A); change in proportion of population becoming infected for varying average node degree (B)

In Supplementary Figure 3 we look at the peak infection rates and the times to stability and peak infection, similarly to Figure 7. Once again, the general trend in the random edge removal case mirrors the one seen in random node removal. The peak infection level decreases as one removes edges, and the rate of this decrease increases as edges are removed. The times to the peak also increase as one removes edges, before decreasing in the extreme cases. The WS and ER networks show similar trends, with the BA being more gradual, with lower peaks but larger regions of change.

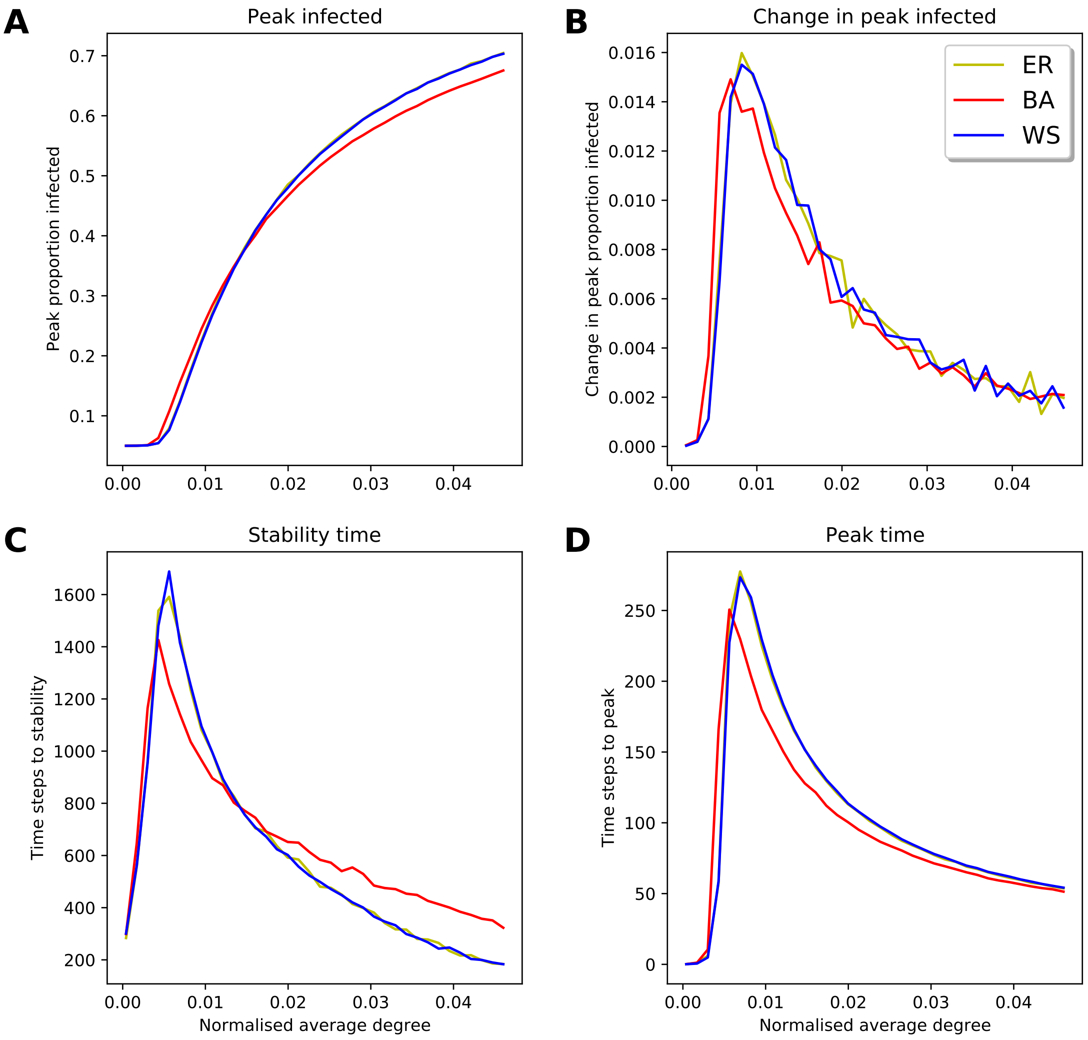

Supplementary Figure 3. Peak infection and stability time for SIR model on synthetic networks with random edge removal. Peak proportion of population infected at same time (A); change in peak proportion of population infected at same time (B); number of time steps to stability (C); number of time steps to peak infection (D)

2.2 Threshold Model

Supplementary Figure 4 replicates Figure 8, showing the proportion of the population becoming infected in the Threshold model as one removes edges. Similarly to the node removal case, initially there is no additional spread; once enough edges have been removed, however, the entire population becomes infected. As before, the ER networks have this change over the smallest region, while the WS networks take place over the largest region.

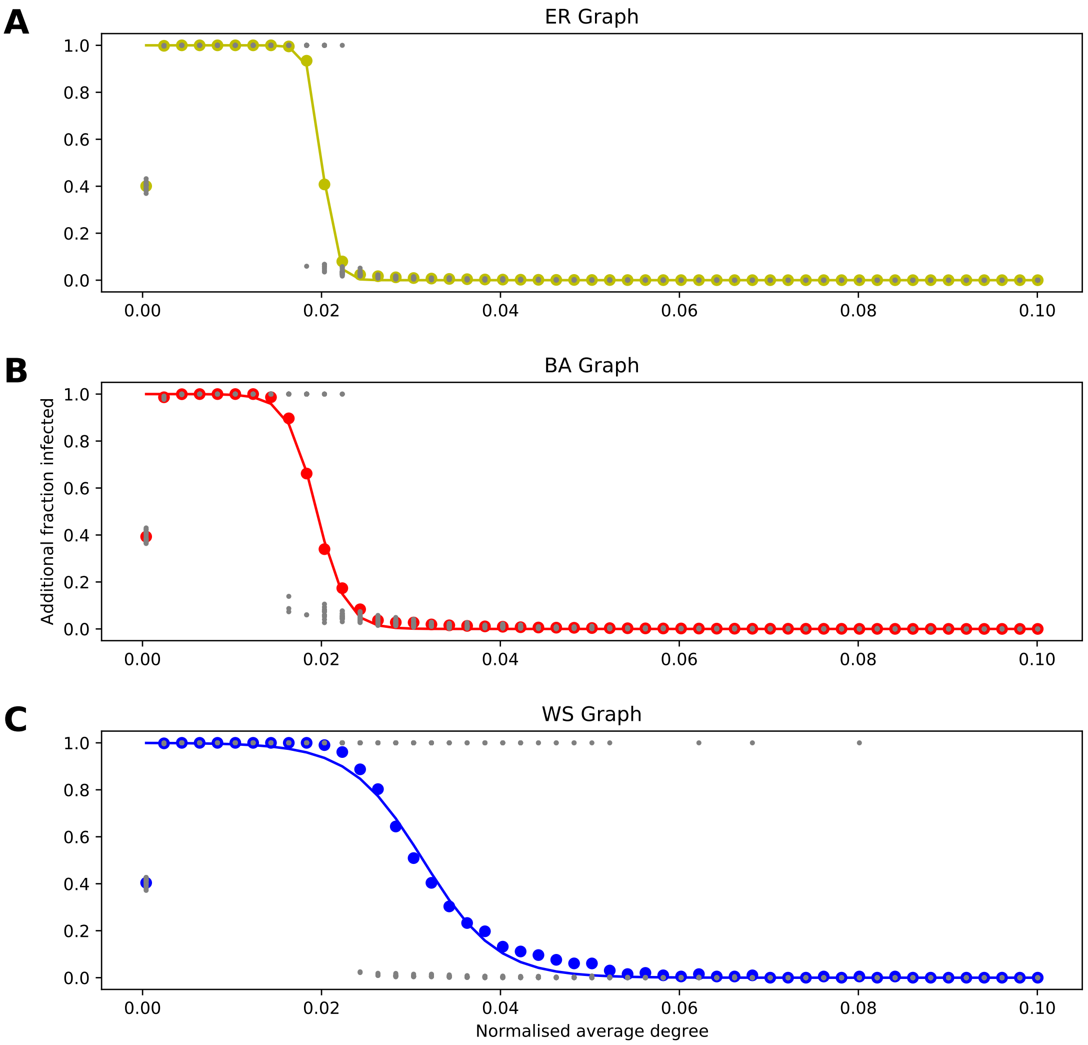

Supplementary Figure 4. Threshold model on synthetic networks with random edge removal. ER network (A); BA network (B); WS network (C)

2.3 Real-World Networks

Supplementary Figure 5 replicates Figure 13, showing how the proportion of the population infected changes as one removes edges. The x-axis is the normalised average degree in the network after the edges have been removed, and the derivatives have been scaled to be relative to 0.0001 and 0.00001 changes in normalised average degree for the *ArXiv* and Slashdot.org networks respectively. Once again, we see the non-linearity of the impact of data handling errors, with similar results as were observed in the node removal on the real-world networks, as well as in the node and edge removal in the synthetic networks.

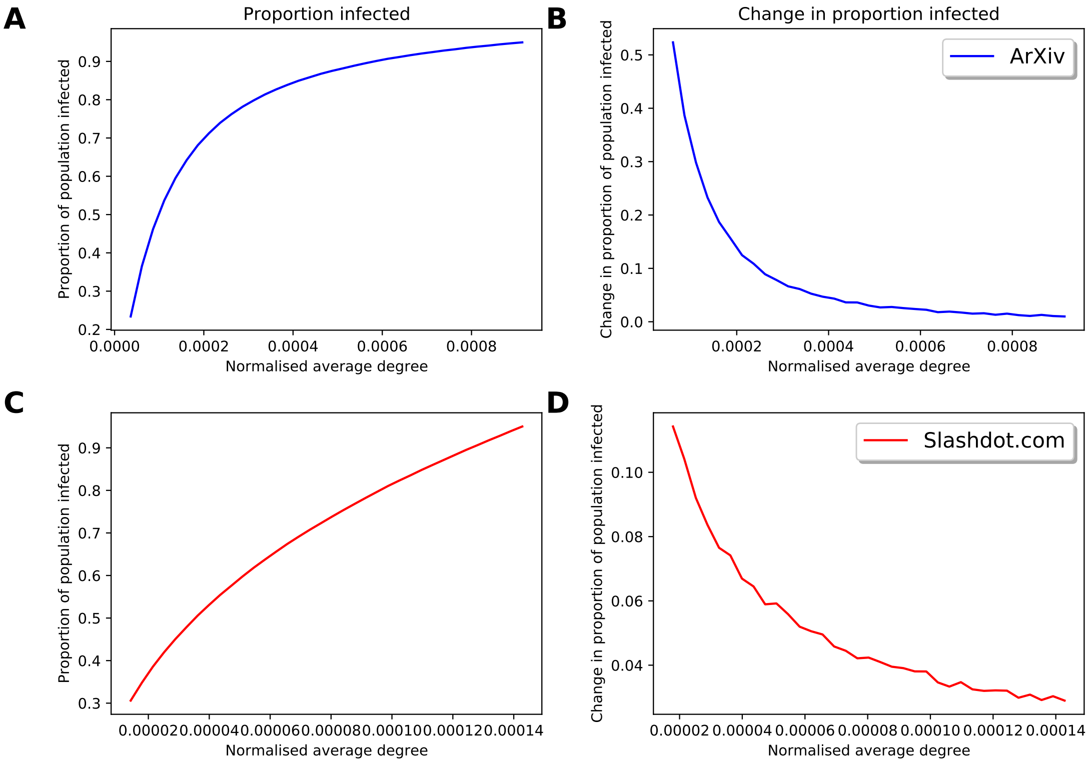

Supplementary Figure 5. Total infection for SIR model on real-world networks with random edge removal. Proportion of population becoming infected for varying average degree on *ArXiv* network and Slashdot.org network (A and C respectively); change in proportion of population becoming infected for varying average degree on *ArXiv* network and Slashdot.org network (B and D respectively)

3 THRESHOLD MODEL ON REAL-WORLD NETWORKS

Supplementary Figure 6 shows the results of running the Threshold model on the real-world networks with the same parameters as were used with the synthetic networks (Figure 8). At first glance, these results seem to differ from those observed in the synthetic networks. We again see non-linear behaviour; now, however, the entire population is initially infected and this infected proportion reduces as nodes are removed. We also see that individual trials no longer only result in full spread or no spread. These differences are most likely a result of the different network sizes and densities. In Figure 8, we note that the curves in the extreme left seem to begin sloping downwards, which could correspond to what is observed in Figure 6.

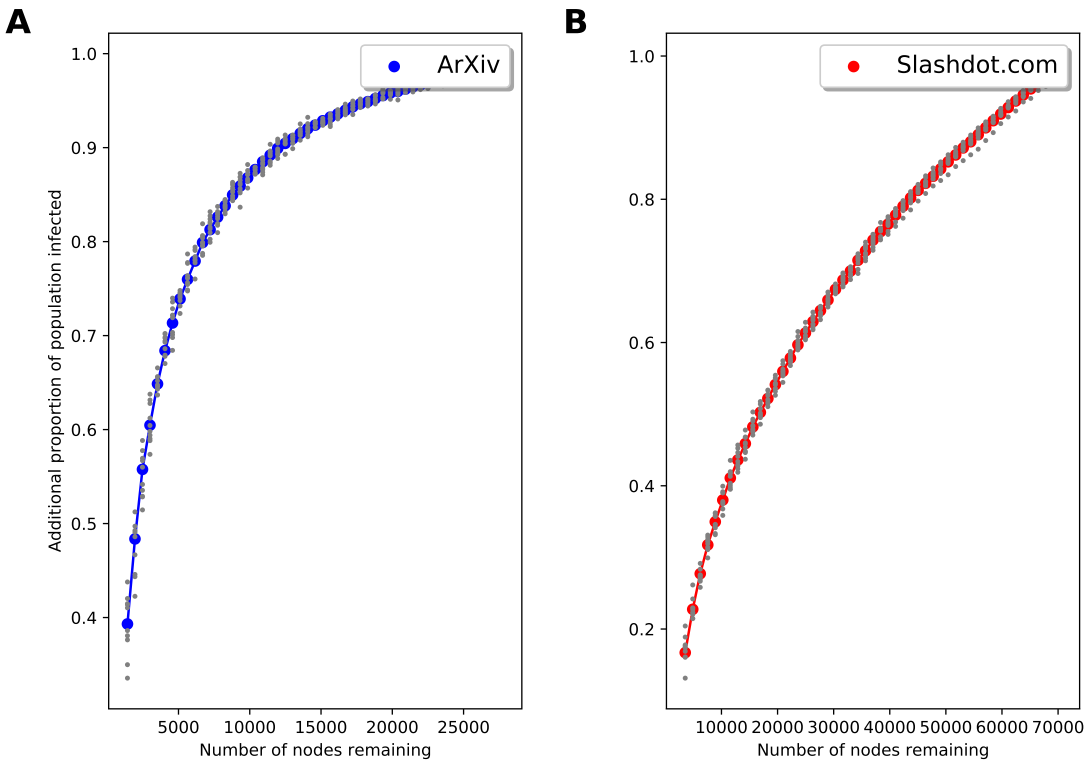

Supplementary Figure 6. Threshold model on real-world networks with random node removal. Additional proportion of nodes infected for varying number of nodes remaining on *ArXiv* network and Slashdot.org network (A and B respectively)

In order to verify this, we run experiments on the synthetic networks removing nodes until only 10 are remaining in each network. Supplementary Figure 7 shows the outcome of the running the model when between 100 and 10 nodes remain in the network, equivalent to a zoomed-in view of the extreme left of Figure 8. Once again, the x-axis represent the number of nodes remaining in the network, and the y-axis the additional proportion of the becoming infected. These results are similar to what is observed in Supplementary Figure 6, providing evidence that the size and density of a network can vary which regions of data handling impact one falls in. For this model, removing nodes from the dense network can increase the spread of a disease, up to a point where the entire population becomes infected. If nodes are continually removed to a point where the network becomes very small, we see the spread decreasing. Alternatively, in a sparse network, removing nodes can reduce the spread of a disease, as seen in the real-world networks. These insights highlight potential areas of further study, such as: what are the network sizes/densities where the behaviour changes, plus how this can vary between different diffusion models.

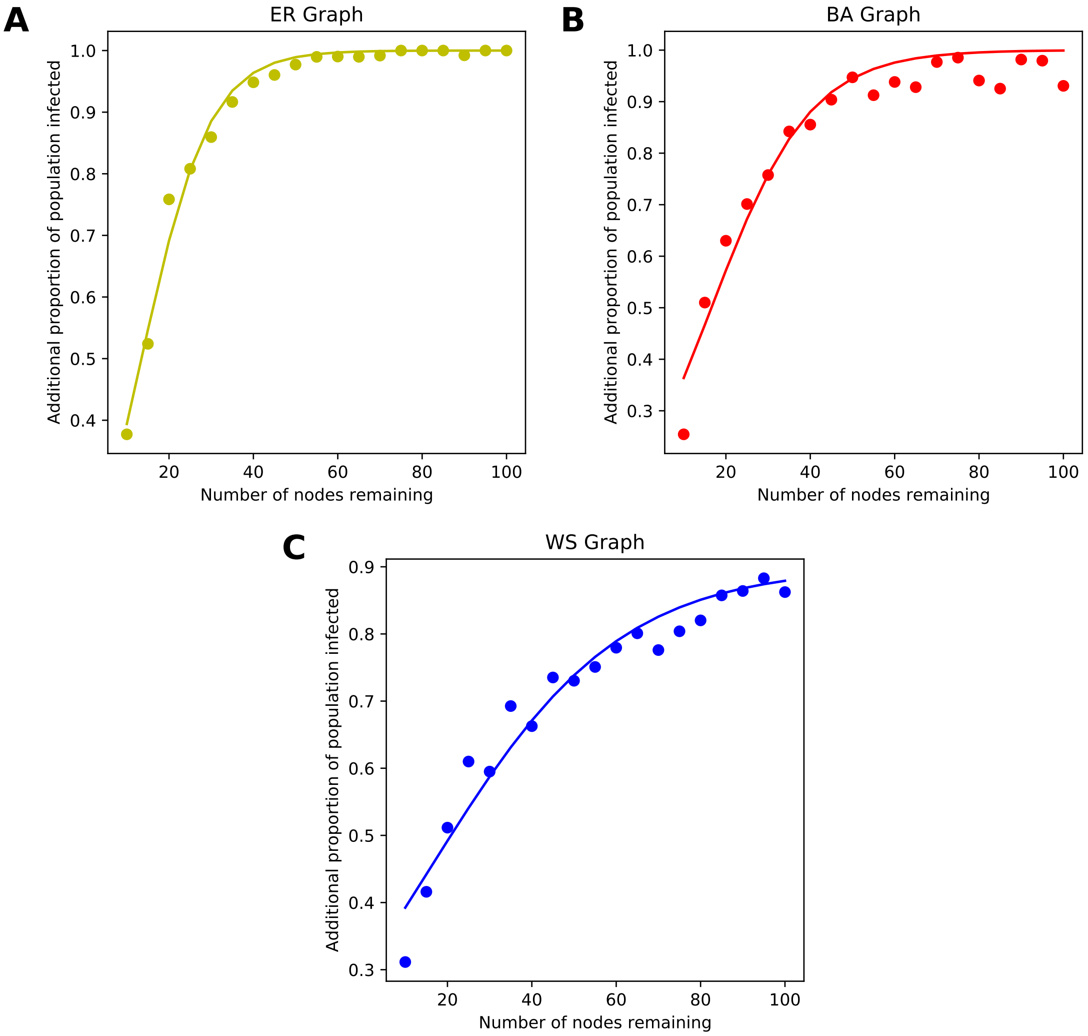

Supplementary Figure 7. Synthetic networks' additional proportion of nodes infected for varying number of nodes remaining. ER, BA, and WS networks (A, B, and C respectively)

4 NODE AGGREGATION ON REAL-WORLD NETWORKS

The node aggregation algorithm applied to the synthetic networks cannot be consistently applied to the real-world networks tested. The real-world networks are assumed to have already been processed through some data handling procedure, for which we do not have the details nor the original raw data. As such, applying the algorithm detailed previously would violate this assumption and result in our having networks that do not reflect real-world networks.

We instead utilise a simple algorithm intended to replicate imperfect data integration. The real-world network is randomly split into two networks with an equal number of edges, intended to represent networks collected from different data sources. These two networks are then integrated, where equivalent nodes appearing in both networks are correctly combined into one node with some accuracy. This is similar in practice to randomly disaggregating nodes by splitting them into two and randomly dividing the edges between them. Algorithm 1 describes the algorithm implemented.

Supplementary Figure 8 shows the result of applying this node aggregation algorithm to the real-world networks and then running the SIR model on them with the parameters used in section 5.3. We see that lowering the accuracy of the node aggregation algorithm leads to a linear increase in the number of node disaggregations, and a linear decrease in the proportion of the population becoming infected. The change in the proportion of the population infected is small – from 0.95 to approximately 0.91. This example illustrates that different data handling methods may not suffer from the non-linearity observed in other

Algorithm 1 Real-world network aggregation simulation

Set aggregation accuracy, α
Divide edge set, E, randomly in half into E_1 and E_2
Define *intersection_nodes* = intersection of nodes in E_1 and E_2
for node i in *intersection_nodes* **do**
    Generate random uniform random variable U between 0 and 1
    if $U > \alpha$ **then**
        Relabel node i in all edges in E_2 to node i_{adj}
    end if
end for
Combine E_1 and E_2 to create one network

cases. Here, the model is fairly robust in both networks to node disaggregation when considering the high-level metric of population infected.

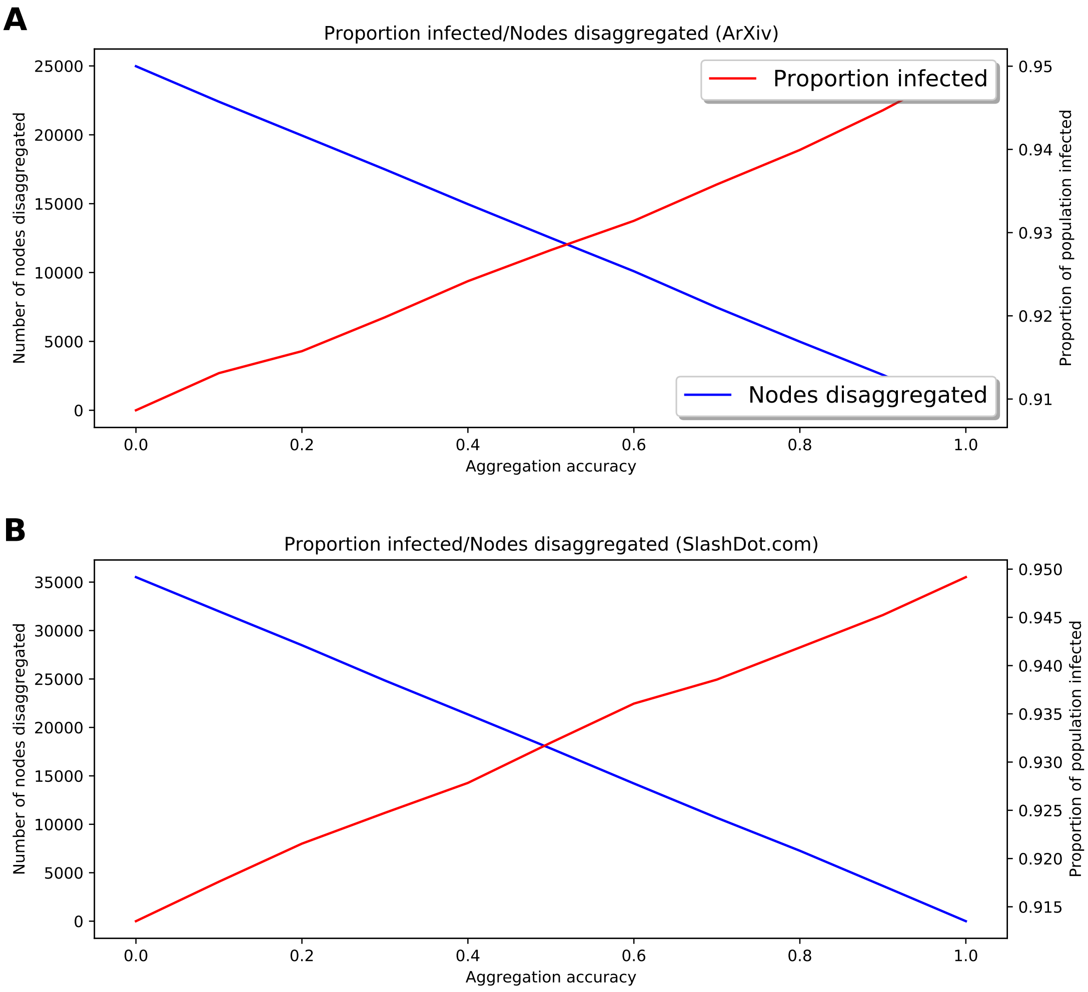

Supplementary Figure 8. Node aggregation and the SIR model on real-world networks. *ArXiv* network and Slashdot.org network (A and B respectively)
