# Supplementary material for: The non-linear impact of data handling on network diffusion models
Source: Patterns (N Y). 2021 Nov 26;2(12):100397. doi: 10.1016/j.patter.2021.100397 (PMC8672192; doi:10.1016/j.patter.2021.100397)
Supplement: Document S2. Article plus supplemental information [file mmc2.pdf]

Article

Patterns

The non-linear impact of data handling on network diffusion models

Highlights

- An overview of basic data handling and common pitfalls
- A framework for analyzing the impact of data handling on network diffusion models
- Results showing the impact of data handling on various standard network models

Authors

James Nevin, Michael Lees, Paul Groth

Correspondence

j.g.nevin@uva.nl

In brief

We identify a critical step in the application of data-driven computational models—data handling. We suggest a framework for testing the impact of data handling and apply it to assess the impact of data handling on various types of network diffusion models. We use our results to highlight the effect that data handling may have on the conclusions researchers draw from models.

Nevin et al., 2021, Patterns *2*, 100397
December 10, 2021 © 2021 The Author(s).
https://doi.org/10.1016/j.patter.2021.100397

CellPress

Patterns

CellPress
OPEN ACCESS

Article

The non-linear impact of data handling on network diffusion models

James Nevin,[1,2,*] Michael Lees,[1] and Paul Groth[1]
[1]University of Amsterdam, Amsterdam, the Netherlands
[2]Lead contact
*Correspondence: j.g.nevin@uva.nl
https://doi.org/10.1016/j.patter.2021.100397

THE BIGGER PICTURE Many computational models rely on real-world data, and the steps required in moving from data collection, to data preparation, to model calibration and input are becoming increasingly complex. Errors in data can lead to errors in model output that might invalidate conclusions in extreme cases. While the impact of errors in data (e.g., in sampling) have been analyzed in the literature, here we highlight the importance of data handling in the modelling and simulation process, and how particular data-handling errors can lead to errors in model output. We develop a framework for assessing the impact of potential data-handling errors for models of spreading processes on networks, a broad class of models that capture many important real-world phenomena, such as epidemics. We apply this framework to test data-handling errors on various standard models. Our results demonstrate that data-handling errors can have significant impact on model conclusions, especially in critical regions of a system.

1 2 3 4 5 **Concept:** Basic principles of a new data science output observed and reported

SUMMARY

Many computational models rely on real-world data, and the steps required in moving from data collection, to data preparation, to model calibration, and input are becoming increasingly complex. Errors in data can lead to errors in model output that might invalidate conclusions in extreme cases. While the challenge of errors in data collection have been analyzed in the literature, here we highlight the importance of data handling in the modeling and simulation process, and how particular data handling errors can lead to errors in model output. We develop a framework for assessing the impact of potential data errors for models of spreading processes on networks, a broad class of models that capture many important real-world phenomena (e.g., epidemics, rumor spread, etc.). We focus on the susceptible-infected-removed (SIR) and Threshold models and examine how systematic errors in data handling impact the predicted spread of a virus (or information). Our results demonstrate that data handling errors can have significant impact on model conclusions especially in critical regions of a system.

INTRODUCTION

The modern computing revolution has led to data science techniques, and in particular computational modeling, being applied in a wide range of fields including sociology,[1] psychology,[2] chemistry,[3] and physics.[4] The influence of computational models can also be felt in society, for example, with the ongoing COVID-19 crisis. In the last year, epidemiologists and modelers have used models to predict the spread of this virus, as well as to test the effectiveness of various measures, such as vaccinations and isolation, on controlling this spread. Governments are relying on models to help make decisions that affect every citizen's way of life.[5]

Inherent in the modeling process is the principle of abstraction, a process that aims to condense processes and phenomena into their most basic ingredients. The dangers and risk of modeling have long been understood[6] and it is vital that modelers explicitly describe their assumptions and reasoning so that models are used in the right way to understand the right phenomena.[7]

It is not just the underlying assumptions that modelers need to take into account. The modeling process has inherent uncertainties and errors that can influence the model and its subsequent predictions. In general, errors can originate at all stages in the modeling and simulation dataflow process. Figure 1 shows a general data pipeline for the process of executing a data-driven

This is an open access article under the CC BY license (http://creativecommons.org/licenses/by/4.0/).

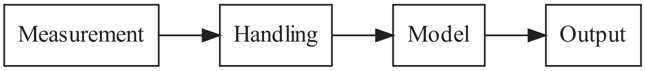

Figure 1. General data pipeline for the process of executing a data-driven computational model

computational model. First, the relevant data are produced through *measurement*—this can be done using, for example, surveys or instruments. Frequently, measurement may not be performed by the modeler—instead they may reuse existing data. Second, the various data sources need to be brought together, cleaned, integrated, and processed. We refer to this as *data handling*. These data can then be used as input to, and to build, a *model*, which can be run to produce its *output* results. Clearly, the quality of a model's output requires the data to be as accurate as possible and hence a minimization of error is desirable. However, total elimination of error is not a realistic or worthwhile objective; instead, it is important to understand and quantify how error propagates through this pipeline to provide some indication of the certainty or reliability of the model output.

Such quantification is a well-studied topic in particular for the measurement and modeling steps. There are a wide variety of techniques for determining the influence of data measurement errors on models and their outputs.[8] This also holds for errors introduced at the modeling stage. Techniques, such as uncertainty quantification,[9] sensitivity analysis, and methods for verification and validation,[10] provide ways to analyze these errors in a quantitative way. These methods describe systematic approaches to understand how accurately a model describes the phenomena of interest, how that accuracy changes with changes in parameter values, and how errors propagate from errors in the model input (data) to errors in the model output.

That being said, the impact of the data handling step on models and their output has not been explicitly considered in the literature. This despite the fact that data handling has become ever more common (through data reuse and sharing) and complex (larger more integrated datasets). Furthermore, large amounts of data are being shared and reused in contexts outside of that for which they were designed. Many of these data are also not well documented, and handling errors can be compounded as one repurposes the same dataset numerous times.[11]

Hence, in this paper, we *draw attention to the potential errors that can be introduced in the data handling step, and how they can influence model output*. We argue that explicitly treating errors in data handling is important for two reasons. First, data handling and the common pitfalls therein, lead to particular types of errors (e.g., incorrect aggregation) that may generate errors that are more systematic compared with measurement errors. Second, by understanding these errors explicitly, we may be able to develop or adapt existing techniques to minimize their impact on the eventual model output.

We consider these errors within the context of models of networks, and, in particular, models of spreading processes (epidemics) and opinion formation (i.e., network diffusion models). While this represents a subset of potential models, it is one where the impact of these data handling errors is more easily understood. Our findings can be extended to data-driven models in general.

The contributions of this paper are as follows:

1. A framework for quantitative analysis of the impact that data handling errors can have on network diffusion models.
2. Results showing the impact of data handling errors on several standard network models. The results demonstrate that models can be impacted in a non-linear fashion and that data handling errors' impact on network structure is not necessarily an indication of subsequent changes in model output.

The remainder of this paper is structured as follows: "background and related work" highlights work on the impact of measurement error on networks; "basics of data handling and common pitfalls" introduces the basics of data handling and how it applies to networks; "methodology" describes our framework for evaluating the effect of data handling on network models, and the parameters and models used in our simulations; "results" details the results of our simulations. Finally, in "conclusions", we summarize our findings.

Background and related work

Before describing the related work, we briefly introduce the network terminology used in this paper.

Network terminology

A network is a graph, **G**, made up of a pair $(\mathbf{V}, \mathbf{E})$, where **V** is a set of vertices (nodes), and **E** is a set of paired vertices called edges (links).[12] The links in a network can be directed or undirected. When applied to real-world systems, nodes represent entities and edges represent links between those entities. For example, if modeling the spread of a virus transmitted through contact, a network can be constructed with nodes representing persons and edges between them representing contact.

We list here some key basic network properties:

- We can define the number of nodes N, and the number of links L. Generally, one might label one's nodes as $i = 1, 2, 3, \ldots, N$
- The degree of a node is the number of links it has to other nodes. For directed links, we can differentiate between the in-degree (number of links that point to the node) and out-degree (number of links that start at the node and point to another node). Henceforth, we limit our definitions and study to networks with only undirected links, known as undirected networks
- We can label the degree of the ith node as k_i. The average degree of a network, $\langle k \rangle$, is the average degree of all the nodes $= \frac{2L}{N} = \frac{1}{N}\sum_{i=1}^{N} k_i$
- Degree distribution, p_k, is the probability that a random node in the network has degree k
- The neighborhood of a node i, $N(i)$, is the set of nodes to which i has a link

When considering network models and data handling, the network structure is often constructed from the data itself. Thus, errors in measurement and handling can propagate through incorrect network structure.

Measurement error and networks

The impact of measurement error (mistakes in the collecting or coding of a network dataset[13]) on network properties has been

well studied. These measurement errors can result in false-negative (missing) nodes and edges, false-positive (should not be there) nodes and edges, and falsely aggregated and disaggregated nodes.

Wang et al.[13] simulated different measurement errors on real-world networks and analyzed their impact on the properties of the networks, such as degree centrality, clustering coefficient, and eigenvector centrality. Smith and Moody[14] and Smith et al.[15] performed similar analyses, also simulating measurement errors and studying their effect on network parameters. These studies concluded that measurement errors can have significant impact on network parameters, and that this impact can be dependent on the structure and properties of the network.

All of these studies focused on the impact of data measurement errors on network properties and not on the data handling step. In addition, these papers consider only the impact of the errors on metrics of network structure and not model output.

We differentiate from this related work in two ways. First, we consider errors arising during the data handling step of executing a data-dependent model. When viewed exclusively through the lens of impact on the network, some of these errors might look identical to measurement errors (false-positive/negative nodes and edges); however, the systematic nature of the errors can be vastly different. Often, modelers will have limited agency in how the data measurement is done, while they may have more control of the data handling step (for example, by changing how they clean and integrate data).

Second, we focus on how these errors can impact models run on the network, rather than simple structural metrics of the network. To do this, we execute simulations using synthetic and real-world data, creating networks of various topologies. The impact of these errors will differ according to the network's topology, model parameters, and the model itself.

Basics of data handling and common pitfalls

In this section, we introduce the key approaches to data handling, focusing on data cleaning and data integration in particular. We detail some of the most common issues that arise in the data handling process and describe how data issues can affect networks.

Data cleaning

There are numerous data issues that need to be dealt with between the collection of the data and supplying it to a model.

One of these primary issues is data cleaning. Abedjan et al.[16] offer an overview of the state of the art in data cleaning methods, and an analysis of various techniques on numerous real-world datasets.

Data cleaning is the process of identifying and correcting data errors. Data errors are defined as deviations from the ground truth: given a dataset, a data error is an atomic value (or a cell) that differs from its given ground truth.[16] Four types of error are identified:

1. Outliers: data values that deviate from the distribution of values in a column of a table
2. Duplicates: distinct records that refer to the same real-world entity. If attribute values do not match, this could signify an additional error
3. Rule violations: values that violate any kind of integrity constraints, such as "not null" or uniqueness requirements
4. Pattern violations: values that violate synthetic and semantic constraints, such as alignment, formatting, misspelling, and semantic data types

The outlier issues are quantitative in nature, while the other three are qualitative. Numerous data cleaning tools exist to address these concerns. They can generally be categorized as: rule-based detection algorithms (such as NADEEF[17]); pattern enforcement and transformation tools (OPENREFINE[18], Data Wrangler[19]); quantitative error detection algorithms; and record linkage and de-duplication algorithms (Data Tamer[20]). However, these tools tend to have their own problems:

- Most algorithms are evaluated on synthetic data or real-world data with synthetically injected errors. This makes it difficult to assess how effective they are in the real-world. In addition, there are few, if any, real-world dirty data with a known ground truth or some sort of universal data cleaning benchmark.
- Real-world data often have multiple types of error. The tools often focus on particular types of error, and there are many tools that address each error, making the process of tool-selection more difficult.
- Many enterprises involve humans in the process of data cleaning. Since human capital is more expensive/limited than computational capital, it is becoming increasingly important to automate the data cleaning process as much as possible.

Abedjan et al.[16] highlight how these tools and problems impact real datasets. There is no dominant tool to solve all data cleaning issues; different tools work well on different datasets, and typically a combination of tools is necessary to achieve good coverage of errors. In addition, the distribution of errors can vary significantly from one dataset to another, meaning the optimal combination of tools will not necessarily always be the same. Adding to this challenge, the ordering in which the tools are applied also has an impact on data cleaning effectiveness, and this will also vary based on dataset. Even with all of these factors optimized for a particular dataset, the coverage of errors is still well below 100%, with the algorithms missing errors that could be detected by humans. Tools such as "openclean"[21] are currently being developed to address these issues.

Data integration

Dong and Srivastava[22] and Doan et al.[23] offer overviews of data integration. Data integration is the process of creating a single, unified view of multiple data sources. There are three common difficulties, which arise sequentially:

- Semantic ambiguity: differences arise in the attributes modeled in various sources. An example of this may be flight takeoff times versus flight gate departure times modeled by different airlines
- Instance representation ambiguity: the same instance or attribute is represented differently by different sources, such as modeling an ID with only digits versus with alphanumerics

- Data inconsistency: different sources report different values for the same attribute

These problems become even more challenging as the number of data sources and the quantity of data provided by each increases. Many data-dependent models utilize data from a variety of sources and in ways in which the data were not originally intended, drastically increasing the likelihood and severity of these issues.

To perform data integration, Dong and Srivastava identify three basic steps: *schema alignment*, *record linkage*, and *data fusion*.

Schema alignment is the process of creating a mediated scheme of all relevant attributes and a mapping from each source to this mediated scheme.

Record linkage identifies which entries from one or many sources correspond to the same entity. It does so by comparing two records and deciding whether they refer to the same entity (termed pairwise-matching). This technique could be rule based (such as some ID attribute needing to be the same), classification based (training a classification algorithm using examples), or distance based. Importantly, with large amounts of data it becomes computationally infeasible to perform matching between all possible pairs of records; thus, blocking algorithms are employed to partition the records before comparison.

Data fusion aims to address differences in reported values for records that refer to the same entity. In brief, a common approach is to allow sources to "vote" on what the true value for the attribute is, with voting repeated iteratively until convergence, or until a certain number of iterations have passed.

Again, we emphasize that in all these steps errors may arise.

Real-world examples

In this section we describe some issues that arise in real-world practice.

Döhmen et al.[24] ran a detailed analysis UK open government data distributed as comma separated value (CSV) files. CSV is one of the most common formats in which data are provided, but can lack potentially crucial information. This can result in incorrect parsing and interpretation decisions, ultimately leading to incorrect downstream decisions.

They identify four categories of common CSV problems

1. CSV Syntax issues: CSVs can have different encodings and different dialects (e.g., what symbol is used as a delimiter), but meta-data on these is not included by default in the file
2. CSV file-level issues: some CSVs do include meta-data, but it is at the top, bottom, or sides of the tables, and thus may need human input to interpret. There are also often empty rows or columns, and files sometimes contain multiple tables
3. Table-level issues: header rows are optional and need to be inferred, with multiple header rows adding to the confusion. Table orientation can vary, and aggregate columns, rows, or cells can disrupt the shape of the table and contain redundant information
4. Column/cell-level issues: CSVs do not support spanning cells, so converting from spreadsheets to CSV may not work correctly. Whitespace is sometimes used, which can impede data type prediction. There are numerous other possible cell-level issues, such as numerics with units, no standard data type encoding (such as dd-mm-yyyy versus mm-dd-yyyy), inconsistent formatting, and confusion over missing values (for example, some CSVs may use special values such as −1 to denote missing values, which the interpreter may not recognize as such)

While common CSV libraries do a good job of parsing and interpreting CSVs, many of these issues can only be resolved through human analysis.

The COVID-19 pandemic has resulted in a multitude of studies highlighting the importance and current lack of effective data handling. Kraemer et al.[25] argue for the necessity of global data curation and standardization in the handling of disease outbreaks. Shankar et al.[26] describe the need for cooperation, communication of data to the public, data privacy, and preparing for the future. Costa-Santos et al.[27] analyze a Portuguese surveillance dataset and identify numerous data quality concerns, and the need for improvement in data management processes. Guidotti and Ardia[28] attempt to offer a unified dataset combining different countries' case numbers and government policies. Davenport et al.[29] highlight how data issues have impacted the handling of the COVID-19 pandemic in the United States. Differences in state reporting make it difficult for decisions to be made at a national level, since they will impact the outcome of the relevant models. These examples highlight the increasing need for effective data handling in addressing urgent concerns.

Data issues on networks

These same data issues can arise when dealing with networks. In general, one wishes to study real-world networks; that is, networks whose nodes and links represent real-world entities and their relationships. For example, people and their friendships. Creating these types of networks requires real-world data. However, as detailed previously, a number of issues can arise in the handling and application of data. These data issues can impact how accurately the network that one constructs reflects reality.

Advani and Malde[30] provide an overview of network data collection and the possible resultant measurement error. They identify six possible sources of measurement error:

1. Missing data due to sampling method
2. Mis-specification of the network boundary
3. Top-coding of the number of edges
4. Miscoding and misreporting of errors (respondent or interviewer error; may forget nodes or become interview fatigued, may report desired rather than true edges)
5. Spurious nodes (spelling mistakes or multiple names may lead to spurious nodes, especially a concern when reusing data)
6. Non-response

They note that different sampling measures can result in different direction and magnitude of error in network statistics. Parameters in economic models on the network can have substantial bias due to mismeasurement of network statistics, and this error is not independent of the true network statistics.

Table 1. Possible resultant errors on networks

Nodes	Edges
Missing	Missing (not present when should be)
Should not be there	Should not be there
Incorrect attributes	Incorrect weighting
	Incorrect direction
	Incorrect type

Networks are fully defined by their links and nodes; these are the two things that can be inaccurate as a result of data issues (Table 1).

Different data handling errors could result in different network errors. For example, incorrect record linkage in data integration could lead to duplicate nodes. In this paper, we focus on the resultant issues in the network, rather than the data-specific cause, but note that *all of these potential network issues can be mapped back to a data handling issue.*

Another important consideration is how the same network error can be a product of a data handling error or a product of a data measurement error.

For example, one might miss nodes in the network due to poor sampling. Alternatively, nodes might be missed due to poor data cleaning leading to the data parser being unable to read the data correctly. The root cause of the issue is different, but the change observed in the network of concern is the same. In both of these cases, the network built using the data is incorrect—it does not accurately reflect the real network (ground truth). It is important that one accounts for these possible errors when one draws conclusions about the network/system as a whole based on the network constructed using the data.

Another consideration is how the cumulative effect on the network, from multiple data issues, could be the same as intentional interventions made to the network. As an example, in modeling the spread of a disease on a network, vaccinating or quarantining a person/node may be modeled through the removal of a node in the network—this node is no longer at risk of being infected, and thus also no longer poses a risk of spreading infection. Likewise, limiting or removing the contact between certain nodes (through, for example, not allowing certain nodes/persons to interact) is the same as removing links in the network. These interventions will often be applied to the network to understand the impact of different interventions. The resultant changes to the network may be equivalent to those caused through data handling errors. The impact of measures, such as immunization and isolation, on networks and diffusion models, particularly for epidemic modeling, have been well studied,[31] and so we can utilize some of these results and analyses, while focusing on a different root cause (data handling errors).

An important part of a network is its robustness. That is, its ability to withstand random failures and attacks in the network. As one removes nodes or edges from a network, one risks "damaging" it—the network may change from connected to disconnected, or some of its properties, such as average degree, diameter, degree distribution, etc., may change drastically. This could have an impact in particular on our understanding of real-world networks, and how processes on them play out.

Percolation theory studies how clusters form on a lattice as one places pebbles with some probability. It shows that there tend to be critical thresholds—points at which the system transitions from many small clusters to one large cluster. What this means for networks, is that removing nodes will initially not break the network up; however, there will be some critical point at which removing just one/a few more nodes will drastically change the network. The greater this threshold (i.e., the greater proportion of nodes that need to be removed for the network to break up), the more robust the network.

Finally, it is important to consider cascading failures. Nodes/entities tend not to fail completely at random; their failure can be influenced by their neighbors. For example, a failure in one link in a power grid will put more strain on the neighboring links, which may lead to more failure. We often want to study how these failures can propagate through the network, which can be done through diffusion models.

Methodology

Figure 2 outlines a framework for testing diffusion model sensitivity to data handling issues. First, graphs are obtained to test with; these graphs could be real-world or synthetic graphs. Following this, a model of data handling is applied to the graph, which results in a set of degraded/inaccurate graphs. Finally, diffusion models can be run on the degraded graphs. The model output on the degraded graphs can then be compared with the output on the original graph, which produces a measure of the sensitivity of the model to the data handling issues.

The framework can be applied to testing the effect of data handling on any diffusion model. One can source synthetic or real-world networks of varying topologies and apply data handling models on top of them before running the diffusion model. This allows for the identification of which data handling errors a diffusion model is most susceptible to, and how network structure interacts with these data handling errors within the context of the diffusion model being considered.

Here, we employ this framework to test the sensitivity of two diffusion models: a susceptible-infected-recovered (SIR)

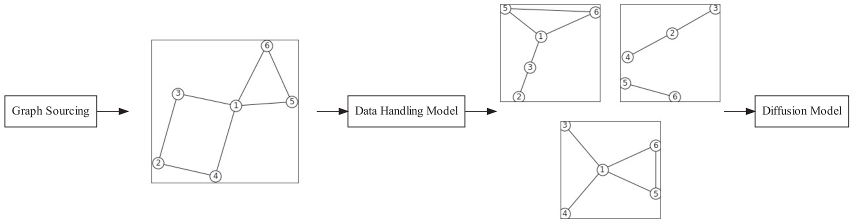

Figure 2. General framework for testing sensitivity of network diffusion models to data handling

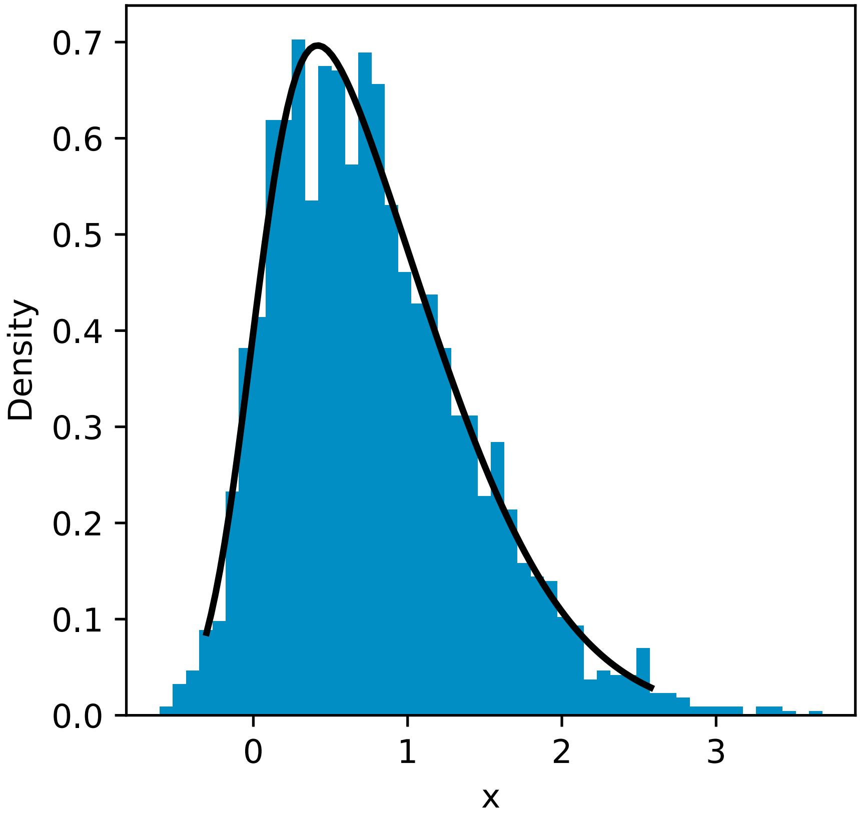

Figure 3. Generated random variables (2,500) from the skew normal distribution with $\alpha = 4$

model and a Threshold model. For our data handling models, we focus in particular on the loss of nodes in the network and the aggregation of nodes based on their neighborhoods. As noted previously, the loss of nodes is something that has been studied in some detail within the context of immunization/isolation and data mismeasurement,[13] but can still help illustrate the importance of dealing with data correctly. The aggregation of nodes has also been analyzed; however, this analysis has been limited to random aggregation, rather than systematic aggregation based on node attributes or properties as is done in this paper.

Graph sourcing

In the first step of the framework, graphs are sourced to test on. These graphs could be created using real-world data, or created synthetically. Here, we focus primarily on synthetic graphs generated using three different algorithms: Erdős-Rényi (ER) graphs, Watts-Strogatz (WS) graphs, and Barabási-Albert (BA) graphs. These synthetic networks aim to reflect a broad class of networks seen frequently in real-world scenarios. The synthetic networks are generated to mimic particular properties of real-world networks, while being easier to study/manipulate. In our analysis, we highlight how data errors and decisions can impact diffusion models in different ways for different network topologies, and the implications this has when deciding upon a data handling approach.

- The ER graph[32,33] has N nodes, and the possible link between each pair of nodes exists with probability p. Alternatively, the number of nodes and edges is specified, and the graph chosen at random from all possible graphs with this number of nodes and edges. Both definitions generate graphs with similar properties. We focus on ones generated through the latter definition
- The WS graph[34] is a graph with the small-world property, meaning random pairs of nodes are not often connected, but will usually share connections with other nodes, and thus have short paths (routes) between them
- The BA graph[35] has the scale-free property, meaning its degree distribution follows a power law, with many low-degree nodes and few high-degree nodes. This results in "hubs" forming in the network—nodes with significantly larger degree than the average

Our experiments follow the design found in Carro et al.[36]: we generate networks with 2,500 nodes, and generate 20 networks per test. The parameters for the networks are chosen so as to have a similar average degree. The ER graphs are generated with 312,500 edges. The WS graphs are generated with each node initially attached to its 250 nearest neighbors, and a rewiring probability of 0.1. The BA graphs are generated with new nodes being attached to 132 other nodes. All networks thus have an average degree of roughly 250, or a normalized average degree of approximately 0.1. The networks are generated using the python package "networkx"[37] (https://networkx.org/).

Data handling model

Once the graph(s) has been sourced, we apply a model of data handling that simulates data handling errors or decisions, resulting in an adjusted network. We simulate two data handling errors/decisions: node removal and node aggregation.

Algorithm 1. Shared neighbor proportion of nodes *i* and *j*

1: *neighbours_i* = array of neighbors of node *i*, not including node *j*
2: *neighbours_j* = array of neighbors of node *j*, not including node *i*
3: *shared_neighbours* = array of elements in both *neighbours_i* and *neighbours_j*
4: Return $\max\left(\frac{|shared\_neighbours|}{|neighbours\_i|}, \frac{|shared\_neighbours|}{|neighbours\_j|}\right)$

CellPress
OPEN ACCESS

Algorithm 2. Systematic aggregation of graph G, with shared neighbor threshold *p* and *K* bins

```
for k = 1, 2, ..., K do
    For nodes i and j in bin k, i ≠ j do
        if Shared neighbor proportion of nodes i and j (Alg. 1) > p then
            Reattach all edges from node j to node i
            Delete node j
        end if
    end for
end for
```

Node removal. To test the effects of data mishandling, we delete nodes at random from the networks. We remove nodes in batches, running our diffusion model on the network after each batch removal. Nodes are removed until 125 nodes remain (this is 5% of the total initial number of nodes, as in Wang et al.[13]), with batch sizes chosen based on our applied diffusion model. We use 35 and 50 batches for the SIR and Threshold models, respectively, with these sizes chosen to balance smoothness of resulting curves and running time.

We note that node removal is also used in the control of epidemics. However, in that case the removals are intentional, whereas here we have unintentional removal due to a data handling error. In addition, the intentional removal is usually not at random; rather, nodes are removed so as to minimize the spread of a virus. As we are focusing on nodes missing due to error, we limit our simulations to missing at random nodes. We also repeat these experiments with random edge removal in "edge removal" of the supplemental experimental procedures, where the results and interpretation are fairly similar to what is observed in the node removal case.

Aggregation. To test the effects of data integration decisions, we simulate the aggregation of nodes using node neighborhoods and randomized attributes. We note that the algorithm detailed below is not intended to replicate perfectly how this might be done in practice, only to illustrate the differences that can arise when one aggregates, whether in some systematic way or with random aggregation.

For each node, we sample a random variable from the skew normal distribution, with probability density function (pdf) given by:

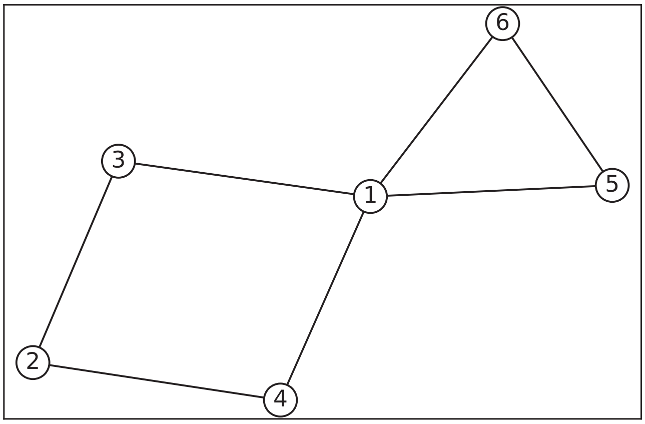

Figure 4. Shared neighbors example graph

$$f(x) = 2\varphi(x)\int_{-\infty}^{\alpha x}\varphi(t)dt, \qquad \text{(Equation 1)}$$

with $\varphi(x) = \frac{1}{\sqrt{2\pi}}e^{-\frac{x^2}{2}}$ (the standard normal pdf). We set $\alpha = 4$, and bin the realizations into 50 bins of equal length. Figure 3 shows an example of this. This distribution and binning emulates entities (nodes) having similar attribute values, where one might check whether they refer to the same real-world entity. We choose a skew distribution because many real-world attribute distributions are skewed.[38] This is a simplification of the entity similarity testing process, but serves to illustrate the effect it may have on network diffusion models. The exact magnitude of the impact made by the aggregation decisions can vary, but we simply aim to show that these decisions can have an impact.

Similarly to the methodology used in Bilgic et al.,[39] we compare the neighborhoods of nodes sharing similar attribute values to the decide whether they refer to the same entity. Nodes are binned together based on their simulated attribute values, with 50 bins of equal length, resulting in bins having between 1 and 160 nodes in each from our 2,500 node networks. The neighbors of any two nodes in the same bin are compared; if the proportion of neighbors in common exceeds some shared neighbor threshold, p, we assume that the nodes refer to the same entity (we ignore direct edges between the two nodes being compared when considering neighbors). Nodes assumed to refer to the same entity are aggregated by deleting one of the nodes and attaching all of its edges to the other node. Algorithm 2 describes this shared neighbor aggregation algorithm, with Algorithm 1 calculating the proportion of neighbors shared.

The proportion of neighbors shared between two nodes may differ based on the degree of the nodes; in this case, we always consider the larger proportion. Figure 4 shows an example of this: suppose we are deciding whether to aggregate nodes 1 and 2 based on their shared neighbors. From the perspective of node 2, 100% of its neighbors (nodes 3 and 4) are shared with node 1; however, from node 1's perspective, only 50% of its neighbors (nodes 3, 4, 5, and 6) are shared with node 2. We will always consider the greater of the two values in these scenarios, and so would aggregate nodes 1 and 2.

We do not address the transitivity issue: pairwise comparisons in each bin are made systematically, with each node being compared once with every other node, and aggregations are done as soon as the conditions are met, without consideration of previous or future pairwise comparison results. Using Figure 4 again for an example, suppose nodes 1, 2, and 5 all belong to the

Algorithm 3. SIR diffusion model

Let State_j denote the state of node j
Set $\text{State}_j = s$ for nodes $j = 1, 2, \ldots, n$
Let $S = \{j : \text{State}_j = s\}$
Define infection rate, β, and recovery rate, γ
Set $\text{State}_j = i$ for initially infected nodes j
Let $I = \{j : \text{State}_j = i\}$
for Number of iterations **do**
  for $j \in I$ **do**
    Let $N(j)$ be the set of neighbors of j
    for $k \in (N(j) \cap S)$ **do**
      Set $\text{State}_k = i$ with probability β
    end for
    Set $\text{State}_j = r$ with probability γ
  end for
end for

same bin, and we use a shared neighbor threshold of 80%. When we compare nodes 1 and 2, we would conclude that they refer to the same entity (since the maximum of their shared neighbors proportion is 100% > 80%); likewise with nodes 1 and 5. However, we would not conclude that nodes 2 and 5 refer to the same entity, since they have no neighbors in common. Thus we have node 1 = node 2, node 1 = node 5, but node 2 $\neq$ node 5. While this is an issue that needs to be dealt with in reality, we avoid addressing it here, and instead simply make aggregation decisions based on the order in which the pairwise comparisons are made. So if we compare nodes 1 and 2 first, we would consider them the same entity and immediately aggregate them (this would essentially delete node 2 and/or relabel node 1 as node 2). We would them compare node 1 with node 5, and would conclude that they are the same and aggregate them. Again, this is a simplification of the entity resolution process but still serves the purpose of emulating how it might affect the network. For each test, we create multiple graphs, which helps address variance concerns arising from this issue.

When running our models on each simulated network, we also consider the network resulting from random node aggregation, as was studied in Smith and Moody.[14] We perform the same number of aggregations as was observed by applying the algorithm above, only now at random.

Diffusion model

Once we have sourced a network and applied a data handling model to it, a diffusion model is run on it. Network diffusion models represent a broad class of models that can be used to model some form of spreading phenomenon. As opposed to traditional diffusion (e.g., on a lattice), the network structure provides a specification of connectivity between the entities and hence defines the pathways by which the spreading (or diffusion) may progress. This spread could represent many different real-world processes: the spreading of failures on a power grid; the spread of a rumor at a party; the spread of a disease through a community.

We are interested in the impact of data on network-based models and how data handling may impact conclusions drawn from these models. While there are other classes of models that can be applied on networks, we restrict our analysis to diffusion models (spreading processes) as they represent a broad class of relevant applications (e.g., epidemics, information spreading, rumor spread, etc.). For succinctness, we primarily use terminology for the spreading of a disease, but the models addressed could equally be applied to any spreading phenomenon.

Algorithm 4. Threshold diffusion model

Let State_j denote the state of node j
Set $\text{State}_j = s$ for nodes $j = 1, 2, \ldots, n$
Let $S = \{j : \text{State}_j = s\}$
Let Thresh_j denote the threshold of node j
Set $\text{State}_j = i$ for initially infected nodes j
Let $I = \{j : \text{State}_j = i\}$
for Number of iterations **do**
  for $j \in S$ **do**
    Let $N(j)$ be the set of the neighbors of j
    if $\frac{|I \cap N(j)|}{|N(j)|} > \text{Thresh}_j$ **then**
      Set $\text{State}_j = i$
    end if
  end for
end for

Graph Sourcing	ER	Nodes: 2 500, edges: 312 500
	BA	Nodes: 2 500, new_edges: 132
	WS	Nodes: 2 500, nearest_neighbours: 250, rewire_prob: 0.1

Data Handling Model	Edge/Node Removal	50 random batches (Threshold), 35 random batches (SIR)
	Aggregation	Shared neighbour threshold: 0.8 (WS) and 0.57 (BA)

Diffusion Model	SIR	β: 0.001, γ: 0.01 (edge/node removal) or 0.2 (aggregation), frac_inf: 0.05
	Threshold	node_threshold: 0.5, frac_inf: 0.36 (edge/node removal) or 0.42 (aggregation)

Figure 5. Summary of simulation parameters and models for synthetic networks

There is a vast array of spreading processes used to model epidemics.[40,41] As stated previously, we focus on two common diffusion processes: the SIR model and the Threshold model.

The SIR model. The SIR model was introduced by Kermack and McKendrick.[42] In this model, each node can be in one of three states: susceptible, meaning they have not yet been infected; infected; and removed, meaning they were previously infected. We assume that removed nodes cannot be infected again, i.e., they become immune to reinfection. The only transitions allowed for nodes is from susceptible to infected, and from infected to removed. Susceptible nodes can become infected when they have contact with other infected nodes.

The model is a Markovian process run with discrete time steps, and can be executed on a network as per Algorithm 3.

The Threshold model. The Threshold model was introduced by Granovetter.[43] In this model, each node/individual has a binary choice/state: either they are infected or not, they believe and spread the rumor or not, they have failed or not. In most cases, we will consider one of the states as positive and one as negative, and study how the number of positive nodes changes over time. For example, we may say an infected node is positive, and study how many nodes become positive in the equilibrium. This model has a susceptible-infected form, with nodes only able to move from the susceptible (negative) to infected (positive) states. Each node has a threshold: the proportion of their neighboring nodes that need to have a positive state for them to switch from the negative state to the positive state. In the power grid example, we might say that a power station fails if 50% of its neighboring stations fail.

Algorithm 4 describes the implementation of this model on a network.

Diffusion model parameters. To illustrate the impact of the data handling model, we run the diffusion models on both the adjusted and the initial networks, and compare the diffusion model outputs. We again follow Carro et al.[36] by using 10 random initializations for every graph and diffusion model tested. Simulations are always run until stability is reached (up to 30 iterations for the Threshold model, and up to 2,500 iterations for the SIR model). All parameter value choices are made for illustrative purposes; we simply wish best to highlight the impact that data handling can have on downstream model output. The impact of the data handling seen when using these models in reality may be greater or lesser than shown here, depending on the data handling performed and the diffusion model chosen.

When testing node removal, we use the following parameters: for the SIR model, β is set to 0.001, γ to 0.01, and the initial fraction infected to 0.05. For the Threshold model, the threshold for each node is set to 0.5, with 0.36 of the population being initially infected. These parameters are chosen simply to illustrate the impact missing nodes might have; a different set parameter values could result in the data mishandling having a greater or lesser impact.

When testing node aggregation, we choose model parameters that result in a roughly 50% spread on the non-aggregated network. We choose these target infection rates as they best illustrate the differences that can arise; in networks or regions where there is extremely high or extremely low spread, how one aggregates is likely to have little to no impact. The 50% case represents scenarios where one's data handling decisions may have the most significant impact. For the SIR model, we adjust $\gamma = 0.2$, and leave $\beta = 0.001$ and 5% initial infection. This results in approximately 50% of the population becoming infected in the non-aggregated network. For the Threshold model, we change to 42% of the nodes initially infected, and leave each node's threshold at 50%. This results in a full spread of the infection in roughly 50% of trials on the WS non-aggregated network.

Algorithm 5. Node removal diffusion

```
Set parameters of diffusion model, M
for Desired number of graphs do
    Generate graph, G, with desired topology
    Run M on G with n random initializations and compute average infection
    for Number of removal batches do
        Remove nodes at random from G
        Run M on G with n random initializations and compute average infection
    end for
end for
```

Algorithm 6. Node aggregation diffusion

1: Set parameters of diffusion model, M
2: **for** Desired number of graphs **do**
3: Generate graph, G, with desired topology
4: Run M on G with *n* random initializations and compute average infection
5: Create a copy of G, G'
6: Bin the nodes of G' into *K* bins using some distribution, *D*
7: Perform systematic aggregation of graph G' (Alg. 2), with shared neighbor threshold *p* and *K* bins calculated previously, and calculate total number of aggregations, *N*
8: Run M on G' with *n* random initializations and compute average infection
9: Create another copy of G, G''
10: Perform *N* random aggregations on graph G''
11: Run M on G'' with *n* random initializations and compute average infection
12: **end** for

Figure 5 summarizes all models, graphs, and parameter values tested in our simulations on synthetic networks. Algorithms 5 and 6 describe our algorithms used for testing diffusion models with node removal and node aggregation respectively.

Génois et al.[44] note that missing nodes correspond to immunization in the network, since a virus cannot spread through, for example, a vaccinated person. Recognizing this, they emphasize that, while the results from the deleting of nodes are the same regardless of why those nodes are deleted, they represent different realities. Nodes removed due to immunization change the real-world network, and thus the diffusion model results on this network are accurate to reality. Nodes removed due to the data being missing, however, lead to an inaccurate network; hence, one can have inaccurate diffusion model results. Génois et al.[44] argue that this difference in cause justifies studying these missing nodes. Because of this, we also include the study of missing nodes, but with a view to understanding missing elements due to data mishandling, as opposed to data mismeasurement as in Génois et al..[44]

Real-world networks

To verify the results on synthetic networks, we also study the impact of node removal on two real-world networks. We use the same networks as were chosen by Wang et al.[13]: a friendship network from Slashdot.org [45] (http://snap.stanford.edu/data/soc-sign-Slashdot081106.html); and a citation network from the e-print repository *ArXiv*[46,47] (http://snap.stanford.edu/data/cit-HepTh.html). As described by Wang et al.,[13] these two networks represent different types of commonly analyzed networks, with similar size but different structural properties, making them good candidates for testing. Similarly to their experiments, we treat the data as ground truth and error free, and transform the networks to be undirected.

For succinctness, we limit our analysis to the SIR model, and provide results for the "threshold model on real-world networks" of the supplemental experimental procedures. When using the model parameters tested on synthetic networks, the spread on the real-world networks without any data handling errors applied is much lower than in the synthetic networks (with approximately 0.3 and 0.6 of the population becoming infected in the Slashdot.org and *ArXiv* networks, respectively). As the region of most concern when applying epidemic models in reality is when one is in a critical region of full spread, we also test SIR model parameters that place the model in these critical regions. We set $\gamma = 0.01$ and the initial proportion of the population infected to 5%. We then adjust the β value to reach regions of critical spread. We note that the two networks used are not connected, so in many cases it may be impossible to infect 100% of the population. Hence, we choose the minimum β value (using 0.005 increments) so that at least 95% of the population becomes infected on average. For the Slashdot.org network, this β value is 0.085; for the *ArXiv* network, it is 0.015. These values correspond to R0 values of 8.5 and 1.5, respectively.

Our node removal simulations in the real-world networks proceed similarly as described in "node removal": nodes are removed in batches until 5% of nodes remain. After each batch removal, the model is run with 10 different random initializations. We follow the experimental design of Wang et al.[13] and perform this procedure with 10 trials on each of the real-world networks. Algorithm 7 describes this process.

Algorithm 7. Node removal diffusion (Real-world network)

1: Set parameters of diffusion model, M
2: **for** Desired number of trials **do**
3: Load real-world graph, G
4: Run M on G with *n* random initializations and compute average infection
5: **for** Number of removal batches **do**
6: Remove nodes at random from G
7: Run M on G with *n* random initializations and compute average infection
8: **end for**
9: **end** for

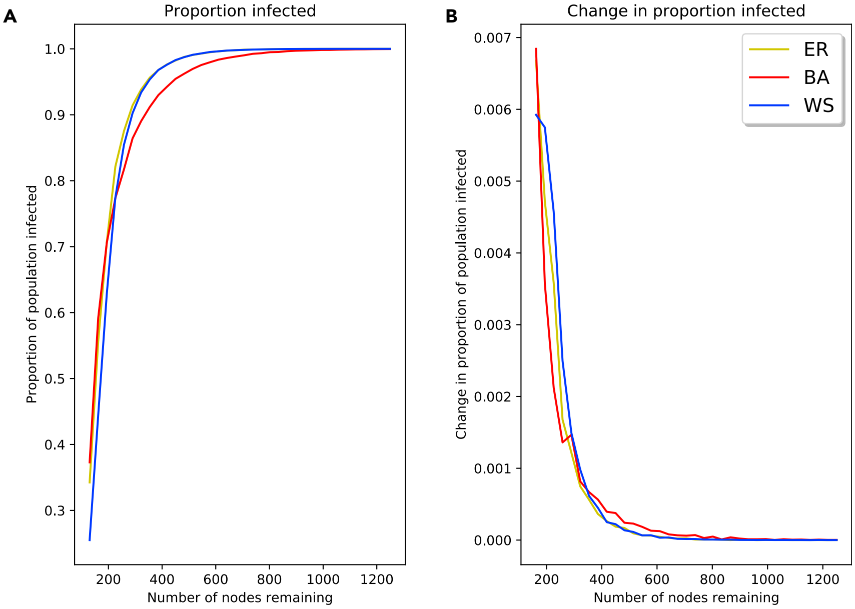

Figure 6. Total infection for SIR model on synthetic networks with random node removal

(A and B) Proportion of nodes infected for varying nodes remaining (A); change in proportion of population becoming infected for varying nodes remaining (B).

RESULTS

We report the results for node removal and node aggregation using the two diffusion models discussed above. As the effects of these types of errors on network properties are well understood ("measurement error and networks"), we focus on the impact on model output, with brief additional results in the "network property changes" of the supplemental experimental procedures. We also note that we consider primarily high-level model metrics, such as proportion of population infected. The conclusions drawn here may differ when studying more detailed metrics.

Node removal

For each of the 20 initial networks of each topology, we ran the model 10 times after each batch of random node removal. In the SIR case, we first removed 1,250 nodes, since the model always had full spread when there were more nodes.

SIR model

Figure 6A shows the proportion of the population becoming infected as one removes nodes at random. We track this relative to the number of nodes remaining in the network. As we remove

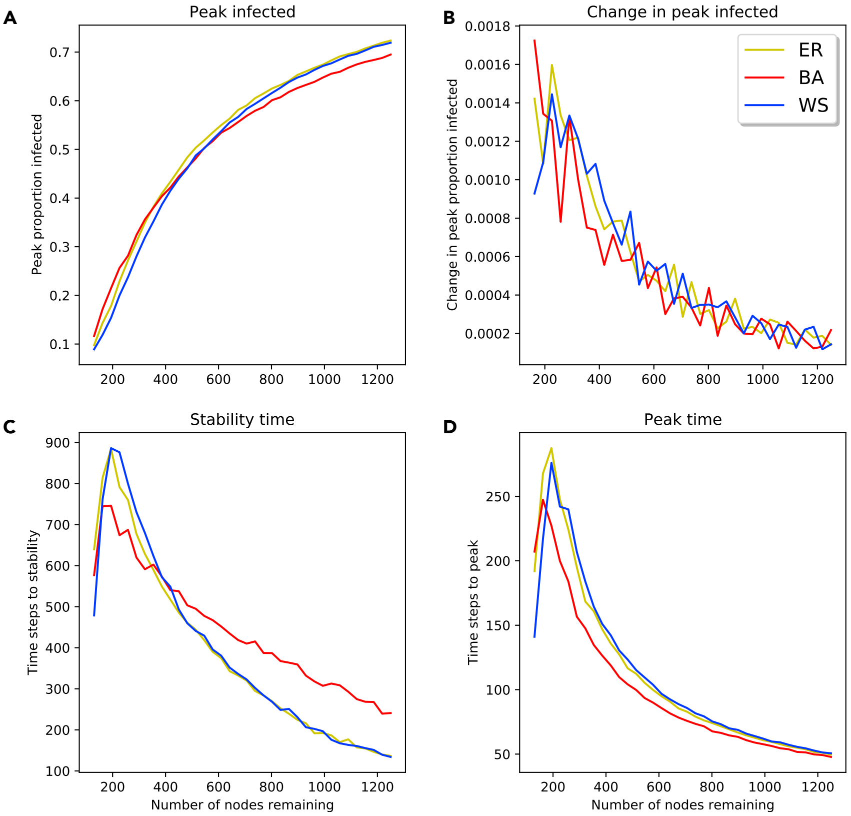

Figure 7. Peak infection and stability time for SIR model on synthetic networks with random node removal

(A–D) Peak proportion of population infected at same time (A); change in peak proportion of population infected at same time (B); number of time steps to stability (C); number of time steps to peak infection (D).

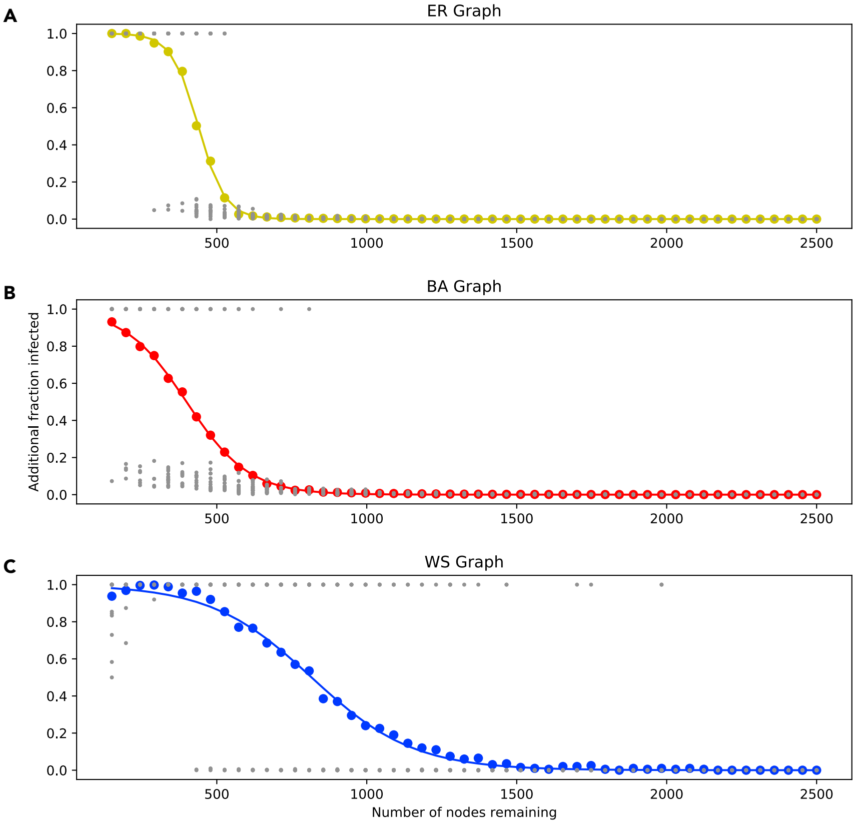

Figure 8. Threshold model on synthetic networks with random node removal

(A–C) ER network (A); BA network (B); WS network (C).

nodes, there is a reduction in the spread of the infection. The rate at which this change in spread happens is almost identical between the ER and WS networks, but differs in the BA network; in the BA network, the spread starts to reduce earlier as one removes nodes, but does not change as rapidly as in the ER and WS networks. In addition, this change in spread is not linear, beginning more slowly as one removes nodes and gradually increasing. This can be seen in Figure 6B, where the change in proportion of the population infected is plotted for varying nodes remaining. We can see that this change is highly non-linear, with an initially slow change that grows as nodes are removed. Again, we note that there are slight differences between the BA network and the ER and WS networks; the BA change graph starts increasing sooner as nodes are removed, but peaks later.

This illustrates an important point: the influence of removing nodes on the model output differs depending on the number of nodes in the network. If one is in a region with many nodes, missing nodes will not have a significant impact on the model output; however, in a region with few nodes, these same missing nodes could have a large impact on the model. This highlights the need for careful data handling in these critical regions. It is also likely that one might find oneself in and around these regions in reality—the usefulness and interest in these models is typically when there is some uncertainty about the potential outcome, since they do not provide much value when one has a large number of nodes and the virus always spreads fully.

In Figure 7, we look at the effect of removing nodes on the peak proportion of the population being infected at the same time and the times taken to reach stability and this peak infection rate. As nodes are removed, the peak proportion of the population infected decreases. Once again, this change is not linear, with a generally sharper decrease at fewer nodes. Both the time to stability and the time to peak infection follow a similar trend, with both generally increasing as nodes are removed, peaking at around 300 nodes; once nodes are removed past this point, the times start to decrease. In both the time measurements and the peak infection rates, we see the trend of the BA network results differing from the ER and WS results. Once again, the BA shows lower peaks and less steep changes, but the impact of node removal begins sooner.

Noticing the non-linear nature of the peak infection rate, we again see the importance of data handling in certain regions. Modelers need to mindful of all steps and possible problems in the modeling process, but especially in their data handling, as this is where they have the most freedom to make changes.

The Threshold model

Figure 8 shows the proportion of the population above the initial infected becoming infected as we remove nodes. The colored plotted points represent the average proportion infected over the multiple trials performed at each number of nodes remaining, with the gray points representing individual trial outcomes; in each individual trial, usually either close to none or close to all nodes become infected. In each plot, a sigmoid function of the form

$$f(x) = \frac{1}{1 + \exp(-k(x - x_0))}, \quad \text{(Equation 2)}$$

is fitted, where k and x_0 are least-squares estimates.

All three scatterplots have the same form, with a high number of nodes resulting in little to no spreading of the disease past the initial infections, and low number of nodes having spread to the entire population. In the WS networks, a very low number of nodes leads to a decrease in the number of infected. In all topologies, there is a transition zone, where one changes from no nodes being infected to all nodes being infected; the length and steepness of this zone varies according to topology. In the ER network, this transition takes place over a small change in number of nodes, while in the WS network it happens over a much larger change.

We see here how the network structure influences the approach modelers need to take to their data handling. In the WS network, there is a large region of nodes remaining where

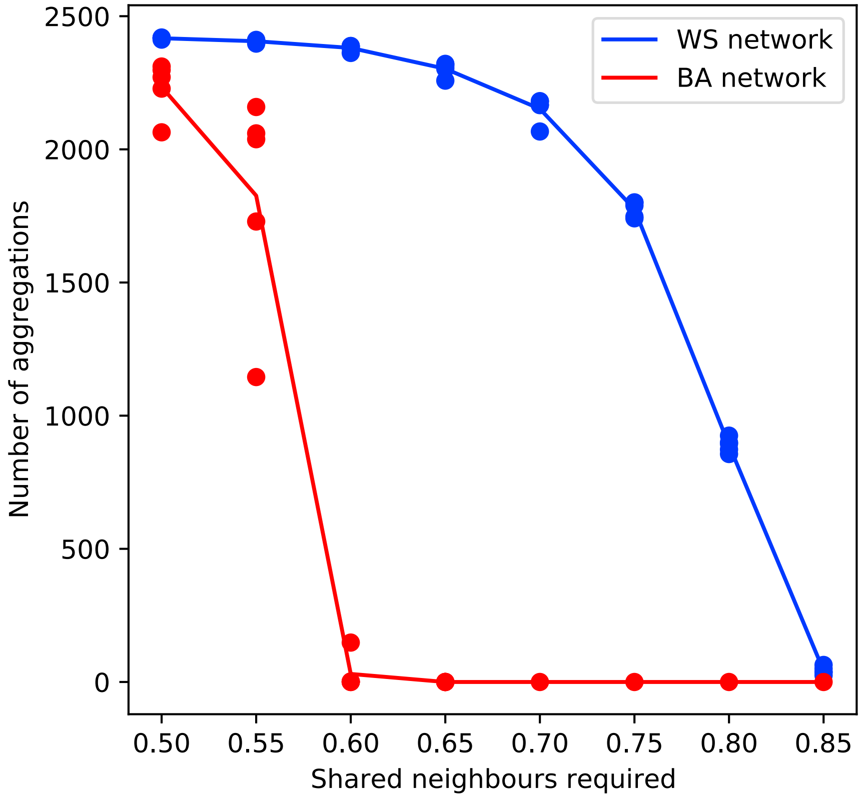

Figure 9. Number of aggregations for various shared neighbor thresholds

small changes can influence the outcome; however, the rate of this change is relatively low. In contrast, in the ER network it is a small region where changing the number of nodes has an impact. In this small region, a change in nodes can drastically shift model outcome. Thus we see that the impact of data handling on model output can be highly network dependent.

Node aggregation

Entity resolution, and thus node aggregation, form critical parts of the data handling process. The modeler is able to make their own decisions in this, with fewer limitations as might be used in data measurement. Here, we show how decisions made can have a greater impact on the downstream model than might be expected based on simply the change in parts of the network structure. We do this through two different decisions: choosing to aggregate nodes using bins and a shared neighbor threshold, and choosing to aggregate nodes at random. This is different from a data measurement issue; we have the full network data, but may have beliefs about nodes representing the same entities, and can decide how or whether to combine them.

We first tested the results of performing the aggregation algorithm prior to running models on the resultant networks. Figure 9 shows the number of aggregations for varying shared neighbor thresholds required to aggregate. The points indicate the results for individual networks, with the lines showing the average over four networks for the two different topologies. For the ER network, aggregations only happened when we set the threshold very low (<0.25), and so we limited our analysis to WS and BA networks only.

The WS and BA networks show drastically different behavior for the given aggregation algorithm. The WS network has a significantly higher threshold (0.8 versus 0.6) where it starts to have aggregations. In addition, there is less variance in the number of aggregations in different graphs for the same shared neighbor threshold, and the change in number of aggregations as one varies the shared neighbor threshold is smoother. When running models on the aggregated networks, we choose a shared neighbor threshold of 0.8 for the WS networks, and 0.57 for the BA networks. These shared neighbor thresholds were chosen to have a moderate number of aggregations. We do not say which of the aggregated or unaggregated networks are the ground truth, if either. We only wish to illustrate how aggregation (with this algorithm in particular) can affect model output.

Table 2 shows the means and standard deviations in the number of aggregations for the shared neighbor thresholds described above, as well as how the aggregation affects the normalized average degree of the networks. These results are for the total of 40 networks used to test the SIR and Threshold models (20 networks each). The chosen shared neighbor thresholds give a similar average number of aggregations, but the BA networks have a much larger variance. Both networks start with a normalized degree of approximately 0.1 (meaning that the average node has 10% of all its possible edges); when one aggregates the nodes using the shared neighbor algorithm, the normalized degree increases slightly (more so for the WS networks); when one performs the same number of aggregations at random, the normalized average degree increases by significantly more. The shared neighbor aggregation does not drastically change the network structure in terms of the average degree measurement, but the random aggregation does. However, both have a significant impact on the outcome of models on the network.

Table 2. Impact of node aggregation on network properties

	Network topology	
	BA	WS
Average number of aggregations	731.175	948.725
Standard deviation of number of aggregations	377.102	105.549
Unaggregated average degree	0.100064	0.100040
Shared neighbor aggregation average degree	0.108332	0.120772
Random aggregation average degree	0.189071	0.220768

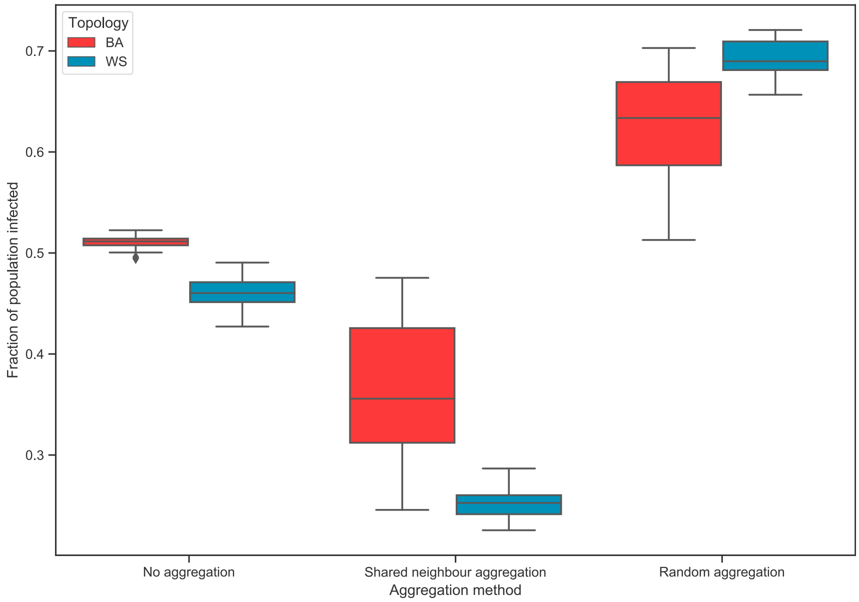

Figure 10. Proportion of population infected for different aggregation techniques (SIR model)

The SIR model

When testing the SIR model with the aggregation algorithm, we choose parameters that result in approximately half of the population becoming infected on the unaggregated network: $\beta = 0.001$, $\gamma = 0.2$, and 5% of the population initially infected. Figure 10 shows the boxplots of the proportion of the nodes becoming infected for different aggregation techniques, while Table 3 shows the means and variances for these proportions. As in the edge removal section, 20 networks were generated for each topology, and the SIR model run 10 times on each with different random initializations. Performing the neighborhood-based aggregation generally leads to a lower overall infection; however, in the BA network there is now far more variance—this can be attributed to the higher variance in the number of aggregations that one has with this network topology. This is similar in the case where one aggregates at random, only now the proportion of the population becoming infected increases.

We see that the neighborhood-based aggregation has a far larger effect on model output than one may suspect based on the changes in the normalized average degree. The directions of the change in the BA and WS networks are the same, but the WS change is of a greater magnitude. The change with random aggregation is also sizeable and of similar magnitude, only in the opposite direction. However, this change is more expected, considering the greater impact seen on the normalized average degree. This shows that the impact of aggregation on model output may not necessarily be known in advance when one only considers its impact on certain network properties, and that how one chooses to do aggregation can lead to drastically different results in model output.

Table 3. Impact of node aggregation on SIR model population infection rates

	Network topology	
	BA	WS
No aggregation	0.510338 (0.000054)	0.460504 (0.000238)
Shared neighbor aggregation	0.368343 (0.00468)	0.250969 (0.000240)
Random aggregation	0.626075 (0.002753)	0.692722 (0.000314)

Proportion infected average values are reported in the "mean (variance)" format.

The Threshold model

For the Threshold model and node aggregation, we chose parameters so that roughly half of trials on the initial WS networks would have no spread, and half would have full spread. This corresponded to each node having a threshold of 50%, and 42% of nodes being infected at initialization, which resulted in roughly 15% of trials on the BA networks having full spread. Figure 11 shows the additional proportion (above the initial infected) becoming infected for different aggregation techniques, with Table 4 showing the means and variances. With the Threshold model, there is more variance in the output when compared with the SIR. The impact of the two aggregation techniques is reversed from the SIR model, with the shared neighbor aggregation resulting in a greater proportion of the population becoming infected, and the random aggregation resulting in a smaller proportion becoming infected.

The results from the Threshold model show that not only does the aggregation technique have an impact on model output but that this impact can also be dependent on the model. In the SIR case, the shared neighbor aggregation reduces the spread, while in the Threshold model it increases the spread.

Real-world networks

Figure 12 shows the proportion of the population becoming infected as we remove nodes from the network. Here, the SIR parameters used are the same as in the synthetic networks: $\gamma = 0.01$, $\beta = 0.001$, and 5% of the population initially infected. In the synthetic networks, this resulted in the population becoming fully infected when no nodes had been removed; instead, in this case either 30% or 60% become infected. The impact of the data handling errors is now fairly linear, unlike what was observed in the synthetic networks. This suggests there may be a certain level of interaction between network properties and model parameters when considering data handling's impact on a diffusion model.

Figure 13 replicates Figure 6 for the two real-world networks with adjusted β values. Shown in Figures 13A and 13C are the

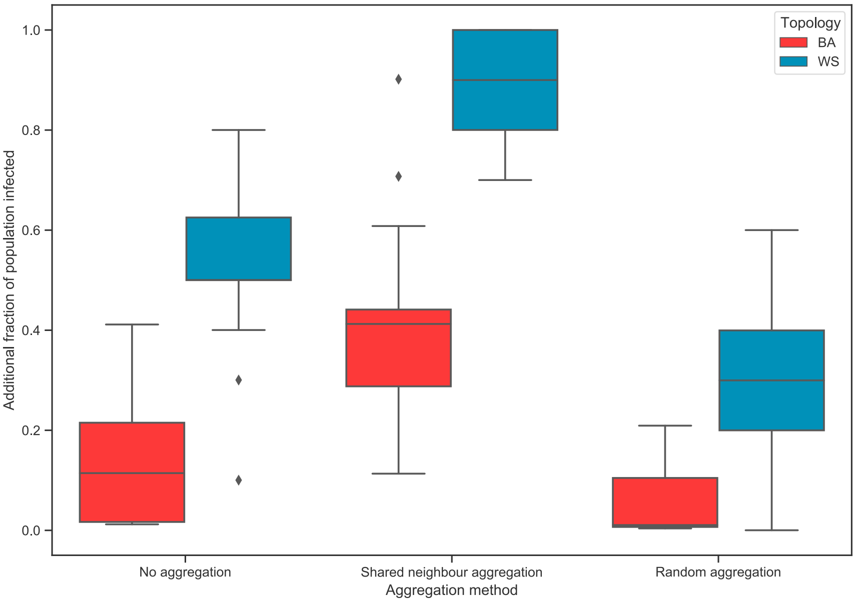

Figure 11. Additional proportion of population infected for different aggregation techniques (Threshold model)

proportions of the population infected as one varies the number of nodes remaining in the network; the change/approximate derivatives of these function are shown in Figures 13B and 13D. As intended, approximately 95% of the population becomes infected when no nodes are removed from the network. We see similar behavior as was observed in the synthetic networks: the change in the proportion of the population becoming infected as one removes nodes is non-linear, with more drastic changes taking place as more nodes are removed. The level of non-linearity differs in the two networks, with the *ArXiv* network showing greater non-linearity. The derivatives of the proportion infected are also different orders of magnitude, with far greater changes in the *ArXiv* network. Part of this can be attributed to the difference in size of the networks (the Slashdot.org network has roughly 2.5 times the number of nodes as the *ArXiv* network), but there are still unexplained differences. Another possible factor in these differences is the density of the two networks: the *ArXiv* network has a normalized average degree 6.5 times that of the Slashdot.org network. The density of both real-world networks is significantly lower than the synthetic networks tested, where we also saw much larger derivative values. While these results do not constitute a proof, they do suggest that network properties, such as density and size, play a role in the effect that data handling errors can have on models.

Table 4. Impact of node aggregation on Threshold model population infection rates

	Network topology	
	BA	WS
No aggregation	0.135021 (0.014993)	0.545207 (0.026795)
Shared neighbor aggregation	0.407204 (0.038125)	0.885116 (0.008692)
Random aggregation	0.044241 (0.003478)	0.295018 (0.024666)

Additional proportion of population infected average values are reported in the "mean (variance)" format.

These results further confirm what was observed in the synthetic networks: the impact of data handling on network diffusion models is dependent on network structure and properties. Again we see that the impact of data handling can be non-linear, and the level of non-linearity is also dependent on the network. This non-linearity also seems more pronounced in regions where modelers may be most concerned in reality, i.e., where there is full spread. While further experiments are required to confirm this, properties such as network size and density may have an effect on the impact of data handling errors. We also see an interaction between diffusion model parameters and data handling error impact.

DISCUSSION

Having highlighted the complexity and concerns of data handling, we provide a framework for testing whether a diffusion model is susceptible to these handling errors. Our framework shows how a modeler can insert data handling errors into networks and run the diffusion model of concern on them, testing the impact on model output. We provide examples by applying this framework to both synthetic and real-world networks, with different data handling and diffusion models applied.

Our results highlight the importance of effective data handling when using data-driven models. In particular, the simulated examples show the quantifiable impact that poor data handling can have. For example, we see that one's perception of the risk-level of a disease turning into a pandemic can be highly dependent on the proportion of the nodes in a network correctly modeled. The outcome of the model changes in a non-linear way as one removes nodes, where removing even small numbers of

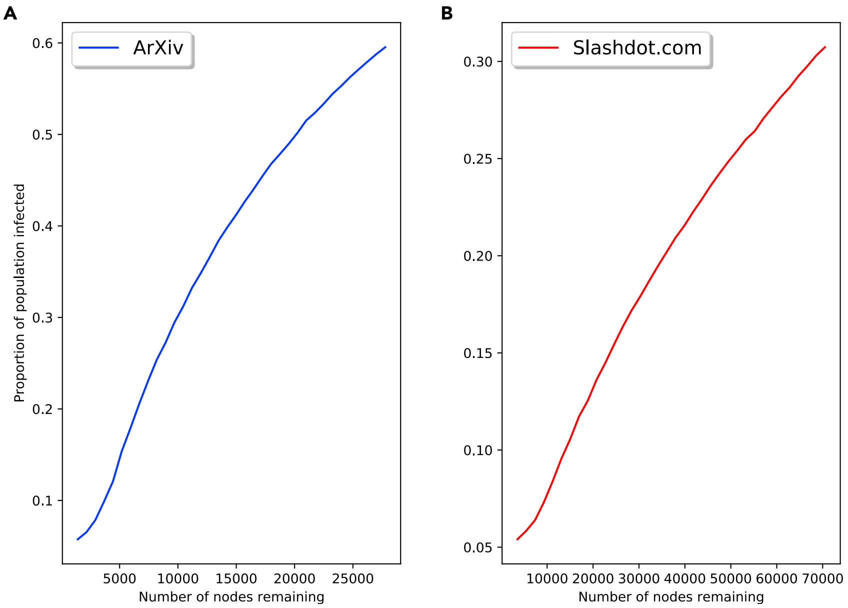

Figure 12. SIR model on real-world network with old parameters

(A and B) Real-world networks' proportion of population becoming infected for varying nodes remaining, using the model parameters tested in the synthetic networks; *ArXiv* network (A); Slashdot.org network (B).

nodes in regions with a few nodes can lead to vastly different predictions for total infections. How severe these changes are is dependent on both the infectiousness of the diseases, and the underlying network structure—both of these need to be taken into account when making decisions based on the available data.

In addition, we show how data integration decisions can have a far-reaching impact on a model's output, and that this impact may not be immediately recognizable based on changes in some network structure properties alone. When aggregating using a shared neighbor approach, there can be very limited changes in the average degree of the network; however, the results of a diffusion model run on this aggregated network can differ significantly from those seen on the unaggregated network. We also show that using random aggregations can have a more noticeable effect on the average degree, but with a similar sized impact on model output. This reinforces the point: data handling decisions can have meaningful impact on model output, and this impact can differ from data mismeasurement impact.

Conclusions

With the increase in the number, diversity, and size of datasets being used for computational modeling, data handling has become increasingly important. This importance will only grow as researchers increasingly reuse data.[48] However, the impact of data handling on computational models is, as of yet, a poorly studied area.

Hence, we introduced a framework for quantitatively analyzing the impact that data handling errors can have on network diffusion models. We used this framework to examine a class of standard network models. We find that:

- the impact of data errors (missing nodes) can create similar impact to simulated interventions (e.g., vaccinations or quarantine in epidemics)
- data handling algorithms, such as data integration, can have non-trivial impact on model output and in some cases conclusions of model runs
- network models (in particular) are highly sensitive to errors in certain connectivity regimes and these regimes are where models transition between qualitatively different outcomes (e.g., full infection versus no infection), hence, the network structure produced by integrating and cleaning data is crucial in these transition situations

There are many avenues for potential research arising from these findings. The devised framework can be applied in various additional ways: on a greater variety of real-world datasets; using more sophisticated, industry-level data integration tools; and on more complex diffusion models. There is also the possibility to develop a set of guidelines for how different types of diffusion models may be affected by data handling, and provide recommendations for how to address potential impacts.

We believe that these findings have important implications for data-driven modelers and again emphasize the important role that data management plays in simulation-based science.

EXPERIMENTAL PROCEDURES

Resource availability

Lead contact

James Nevin is the lead contact of this study and can be reached at: j.g.nevin@uva.nl.

Materials availability

The Jupyter notebook used for running the simulations can be obtained via Mendeley Data repository: https://doi.org/10.17632/fpjpznrbt2.2.

Data and code availability

All original code has been deposited at Mendeley Data: https://doi.org/10.17632/fpjpznrbt2.2, and is publicly available as of the date of publication. This paper analyses existing public data. These data are available at: http://snap.stanford.edu/data/cit-HepTh.html, and http://snap.stanford.edu/data/soc-sign-Slashdot081106.html

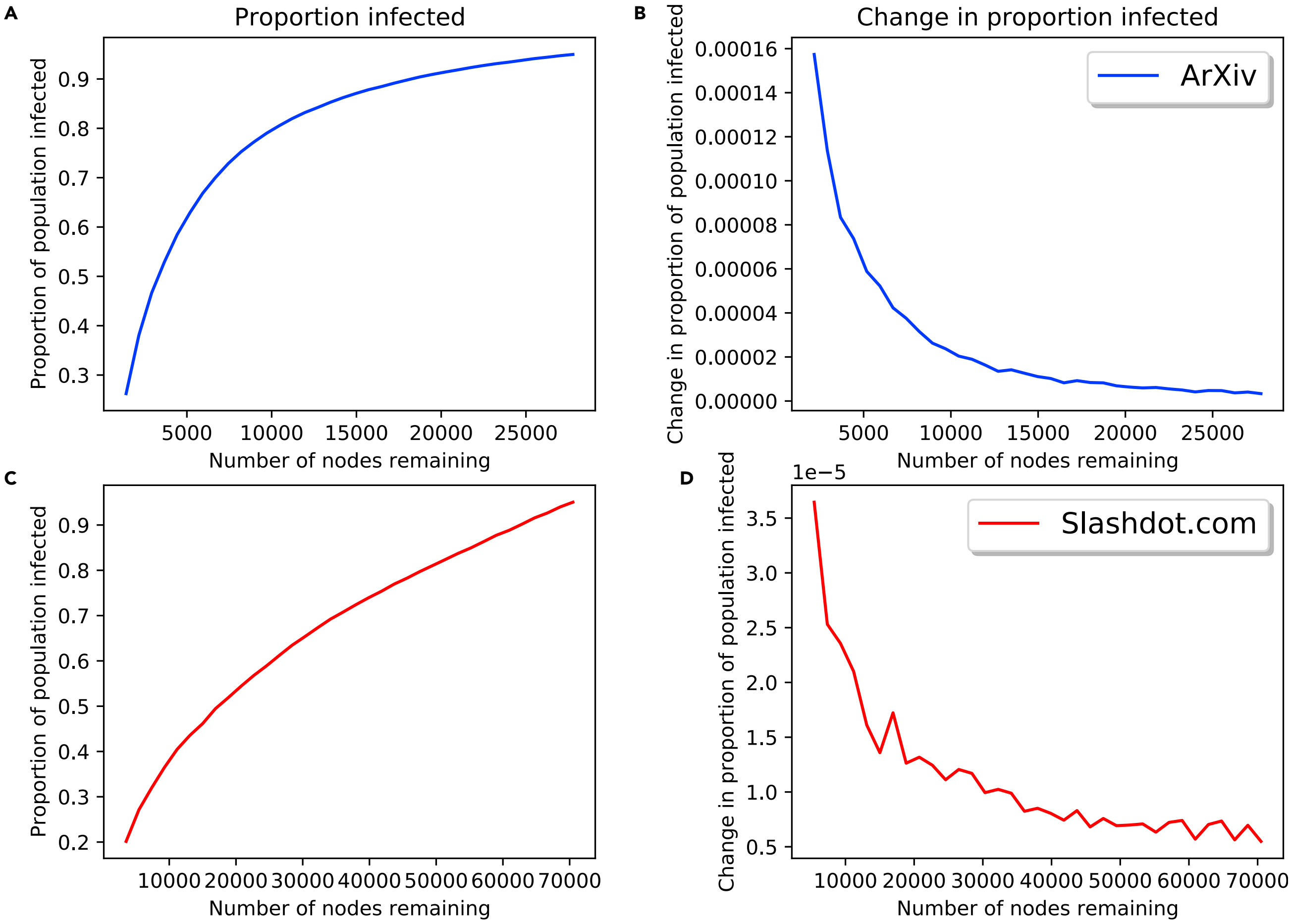

Figure 13. Total infection for SIR model on real-world networks with random node removal
(A–D) Proportion of population becoming infected for varying nodes remaining on *ArXiv* network and Slashdot.org network (A) and (C), respectively; change in proportion of population becoming infected for varying nodes remaining on *ArXiv* network and Slashdot.org network (B and D), respectively.

SUPPLEMENTAL INFORMATION

Supplemental information can be found online at https://doi.org/10.1016/j.patter.2021.100397.

ACKNOWLEDGMENTS

The authors confirm that they have no funders to declare.

AUTHOR CONTRIBUTIONS

Conceptualization, M.L. and P.G.; methodology, J.N., M.L., and P.G.; software, J.N.; validation, J.N. and M.L.; formal analysis, J.N.; investigation, J.N.; resources, M.L. and P.G.; writing – original draft, J.N.; writing – review & editing, J.N., M.L., and P.G.; visualization, J.N.

DECLARATION OF INTERESTS

The authors declare no competing interests.

Received: June 16, 2021
Revised: September 23, 2021
Accepted: November 2, 2021
Published: November 26, 2021

REFERENCES

1. Macy, M.W., and &Willer, R. (2002). From factors to actors: computational sociology and agent-based modeling. Annu. Rev. Sociol. *28*, 143–166.
2. Boden, M.A. (1988). Computer Models of Mind: Computational Approaches in Theoretical Psychology (Cambridge University Press).
3. Jensen, F. (2017). Introduction to Computational Chemistry (John Wiley & Sons).
4. Thijssen, J. (2007). Computational Physics (Cambridge University Press).
5. Adam, D. (2020). Special report: the simulations driving the world's response to COVID-19. Nature *580*, 316–319.
6. Hopkins, J.C., and Leipold, R.J. (1996). On the dangers of adjusting the parameter values of mechanism-based mathematical models. J. Theor. Biol. *183*, 417–427.
7. Rao, C., Wu, Y., Konishi, S., and Mukerjee, R. (2001). On Model Selection. Lecture Notes-Monograph Series (Springer), pp. 1–64.
8. Buonaccorsi, J.P. (2010). Measurement Error: Models, Methods, and Applications (CRC Press).
9. Smith, R.C. (2013). Uncertainty Quantification: Theory, Implementation, and Applications, *Vol. 12* (Siam).
10. Carson, J.S. (2002). Model verification and validation. Proc. Winter Simul. Conf. *1*, 52–58.

11. Pasquetto, I.V., Randles, B.M., and Borgman, C.L. (2017). On the reuse of scientific data. Data Sci. J. *16*, 8.
12. Barabási, A.-L. (2013). Network science. Phil. Trans. R. Soc. A Math. Phys. Eng. Sci. *371*, 20120375.
13. Wang, D.J., Shi, X., McFarland, D.A., and Leskovec, J. (2012). Measurement error in network data: a re-classification. Social Netw. *34*, 396–409.
14. Smith, J.A., and Moody, J. (2013). Structural effects of network sampling coverage I: nodes missing at random. Social Netw. *35*, 652–668.
15. Smith, J.A., Moody, J., and Morgan, J.H. (2017). Network sampling coverage II: the effect of non-random missing data on network measurement. Social Netw. *48*, 78–99.
16. Abedjan, Z., Chu, X., Deng, D., Fernandez, R.C., Ilyas, I.F., Ouzzani, M., Papotti, P., Stonebraker, M., and Tang, N. (2016). Detecting data errors: where are we and what needs to be done? Proc. VLDB Endow. *9*, 993–1004.
17. Dallachiesa, M., Ebaid, A., Eldawy, A., Elmagarmid, A., Ilyas, I.F., Ouzzani, M., and Tang, N. (2013). Nadeef: a commodity data cleaning system. In Proceedings of the 2013 ACM SIGMOD International Conference on Management of Data, pp. 541–552.
18. Ham, K. (2013). Openrefine (version 2.5). J. Med. Libr. Assoc. JMLA *101*, 233.
19. Kandel, S., Paepcke, A., Hellerstein, J., and Heer, J. (2011). Wrangler: interactive visual specification of data transformation scripts. In Proceedings of the SIGCHI Conference on Human Factors in Computing Systems, pp. 3363–3372.
20. Stonebraker, M., Bruckner, D., Ilyas, I.F., Beskales, G., Cherniack, M., Zdonik, S.B., Pagan, A., and Xu, S. (2013). Data Curation at Scale: The Data Tamer System*2013* (CIDR).
21. Muller, H., Castelo, S., Qazi, M., and Freire, J. (2021). Openclean. https://github.com/VIDA-NYU/openclean-core.
22. Dong, X.L., and Srivastava, D. (2015). Big data integration. Synth. Lect. Data Manag. *7*, 1–198.
23. Doan, A., Halevy, A., and Ives, Z. (2012). Principles of Data Integration (Elsevier).
24. Döhmen, T., Mühleisen, H., and Boncz, P. (2017). Multi-hypothesis CSV parsing. In Proceedings of the 29th International Conference on Scientific and Statistical Database Management, pp. 1–12.
25. Kraemer, M.U., Scarpino, S.V., Marivate, V., Gutierrez, B., Xu, B., Lee, G., Hawkins, J.B., Rivers, C., Pigott, D.M., Katz, R., et al. (2021). Data curation during a pandemic and lessons learned from COVID-19. Nat. Comput. Sci. *1*, 9–10.
26. Shankar, K., Jeng, W., Thomer, A., Weber, N., and Yoon, A. (2021). Data curation as collective action during COVID-19. J. Assoc. Inf. Sci. Technol. *72*, 280–284.
27. Costa-Santos, C., Neves, A.L., Correia, R., Santos, P., Monteiro-Soares, M., Freitas, A., Ribeiro- Vaz, I., Henriques, T., Rodrigues, P.P., Costa-Pereira, A., et al. (2020). COVID-19 surveillance—a descriptive study on data quality issues. medRxiv. https://doi.org/10.1101/2020.11.03.20225565.
28. Guidotti, E., and Ardia, D. (2020). COVID-19 data hub. J. Open Source Softw. *5*, 2376.
29. Davenport, T.H., Godfrey, A.B., and Redman, T.C. (2020). To fight pandemics, we need better data. MIT Sloan Manag. Rev. *62*, 1–4.
30. Advani, A., and Malde, B. (2014). Empirical Methods for Networks Data: Social Effects, Network Formation and Measurement Error (tech. rep.) (IFS), Working Papers.
31. Della Rossa, F., Salzano, D., Di Meglio, A., De Lellis, F., Coraggio, M., Calabrese, C., Guarino, A., Cardona-Rivera, R., De Lellis, P., Liuzza, D., et al. (2020). A network model of Italy shows that intermittent regional strategies can alleviate the COVID-19 epidemic. Nat. Commun. *11*, 1–9.
32. Erdős, P., and R′enyi, A. (1960). On the evolution of random graphs. Publ. Math. Inst. Hungarian Acad. Sci. *5*, 17–60.
33. Gilbert, E.N. (1959). Random graphs. Ann. Math. Stat. *30*, 1141–1144.
34. Watts, D.J., and Strogatz, S.H. (1998). Collective dynamics of ‘small-world’ networks. Nature *393*, 440–442.
35. Barabási, A.-L., and Albert, R. (1999). Emergence of scaling in random networks. Science *286*, 509–512.
36. Carro, A., Toral, R., and San Miguel, M. (2016). The noisy voter model on complex networks. Sci. Rep. *6*, 1–14.
37. Hagberg, A., Swart, P., and S Chult, D. (2008). Exploring Network Structure, Dynamics, and Function Using Networkx (Tech. rep.) (Los Alamos National Lab.(LANL)).
38. Simon, H.A. (1955). On a class of skew distribution functions. Biometrika *42*, 425–440.
39. Bilgic, M., Licamele, L., Getoor, L., and Shneiderman, B. (2006). D-dupe: an interactive tool for entity resolution in social networks. In 2006 IEEE Symposium on Visual Analytics Science and Technology, pp. 43–50.
40. Pastor-Satorras, R., Castellano, C., Van Mieghem, P., and Vespignani, A. (2015). Epidemic processes in complex networks. Rev. Mod. Phys. *87*, 925.
41. Nowzari, C., Preciado, V.M., and Pappas, G.J. (2016). Analysis and control of epidemics: a survey of spreading processes on complex networks. IEEE Control Syst. Mag. *36*, 26–46.
42. Kermack, W.O., and McKendrick, A.G. (1927). A contribution to the mathematical theory of epidemics. Proc. R. Soc. Lond. Ser. A, Contain. Pap. Math. Phys. Char. *115*, 700–721.
43. Granovetter, M. (1978). Threshold models of collective behavior. Am. J. Sociol. *83*, 1420–1443.
44. Génois, M., Vestergaard, C.L., Cattuto, C., and Barrat, A. (2015). Compensating for population sampling in simulations of epidemic spread on temporal contact networks. Nat. Commun. *6*, 1–13.
45. Leskovec, J., Huttenlocher, D., and Kleinberg, J. (2010). Signed networks in social media. In Proceedings of the SIGCHI Conference on Human Factors in Computing Systems, pp. 1361–1370.
46. Leskovec, J., Kleinberg, J., and Faloutsos, C. (2005). Graphs over time: densification laws, shrinking diameters and possible explanations. In Proceedings of the Eleventh ACM SIGKDD International Conference on Knowledge Discovery in Data Mining, pp. 177–187.
47. Gehrke, J., Ginsparg, P., and Kleinberg, J. (2003). Overview of the 2003 KDD cup. Acm SIGKDD Explor. Newsl. *5*, 149–151.
48. Koesten, L., Vougiouklis, P., Simperl, E., and Groth, P. (2020). Dataset reuse: toward translating principles to practice. Patterns *1*, 100136.

Patterns, Volume *2*

Supplemental information

The non-linear impact of data handling on network diffusion models

James Nevin, Michael Lees, and Paul Groth

Supplemental Experimental Procedures

1 NETWORK PROPERTY CHANGES

Supplementary Figure 1 shows the impact of node removal on various network properties for the networks tested. The impact is most visible in the closeness centralities, where we see a non-linear impact of node removal. The average clustering coefficient is robust to node removal. The normalised average degree does show some change (especially in the BA network), but these changes are of relatively low magnitude (0.1 to 0.097). These same data handling errors showed non-linear behaviour on diffusion model output, highlighting that data handling's impact on network properties does not necessarily mirror its impact on diffusion models.

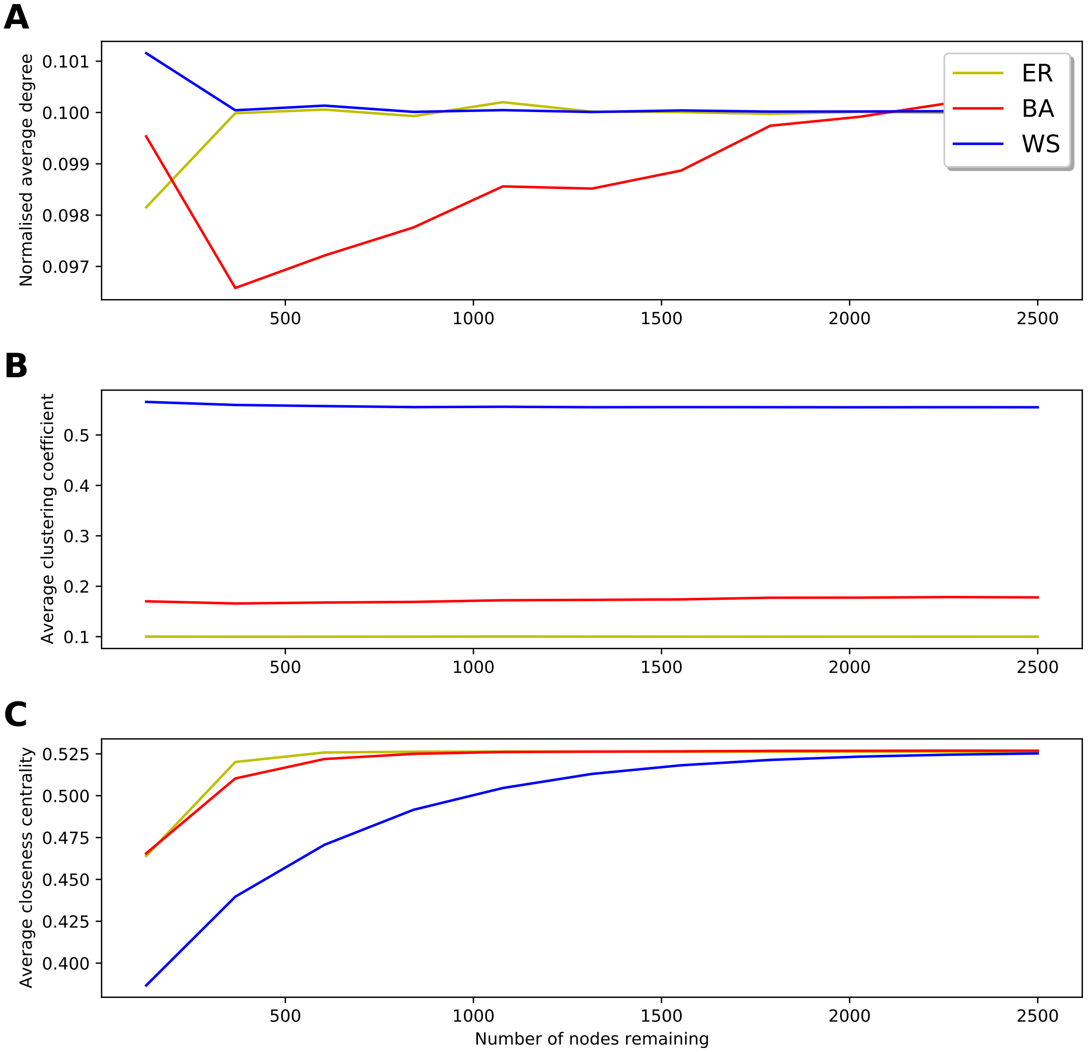

Supplementary Figure 1. Network property changes as nodes are removed. Network normalised average degree (A), average clustering coefficient (B), and average closeness centrality (C) for various nodes remaining

2 EDGE REMOVAL

For each of the 20 initial networks of each topology, we run the model 10 times after each batch of random edge removal. In the SIR case, edge removal is continued until the normalised average degree has been reduced to approximately 0.05 (roughly 50% of edges removed) prior to running multiple iterations of the

diffusion model, as all trials result in full spread of the infection at normalised average degrees above this. The results of random edge removal are generally very similar to those seen in random node removal when using high-level metrics.

2.1 SIR Model

Supplementary Figure 2 replicates Figure 6 for edge removal, showing the proportion of the population becoming infected when running the SIR model (and its change) as we vary the normalised average degree of the networks. As we remove edges and thus lower the average degree of the network, there is a reduction in the spread of the infection. The rate at which this change in spread happens is almost identical between the ER and WS networks, but differs in the BA network; in the BA network, the spread starts to reduce earlier as one removes edges, but does not change as rapidly as in the ER and WS networks. Additionally, this change in spread is not linear, beginning more slowly as one removes edges and gradually increasing. This can be seen in the change plot (this change has been scaled so that it represents the change relative to a change of 0.001 in the normalised average degree). We can see that this change is highly non-linear, with peaks observed at an average degree of around 0.01. Again, we note that this differs between the BA network and the ER and WS networks; the BA change graph has a lower peak with a less steep gradient.

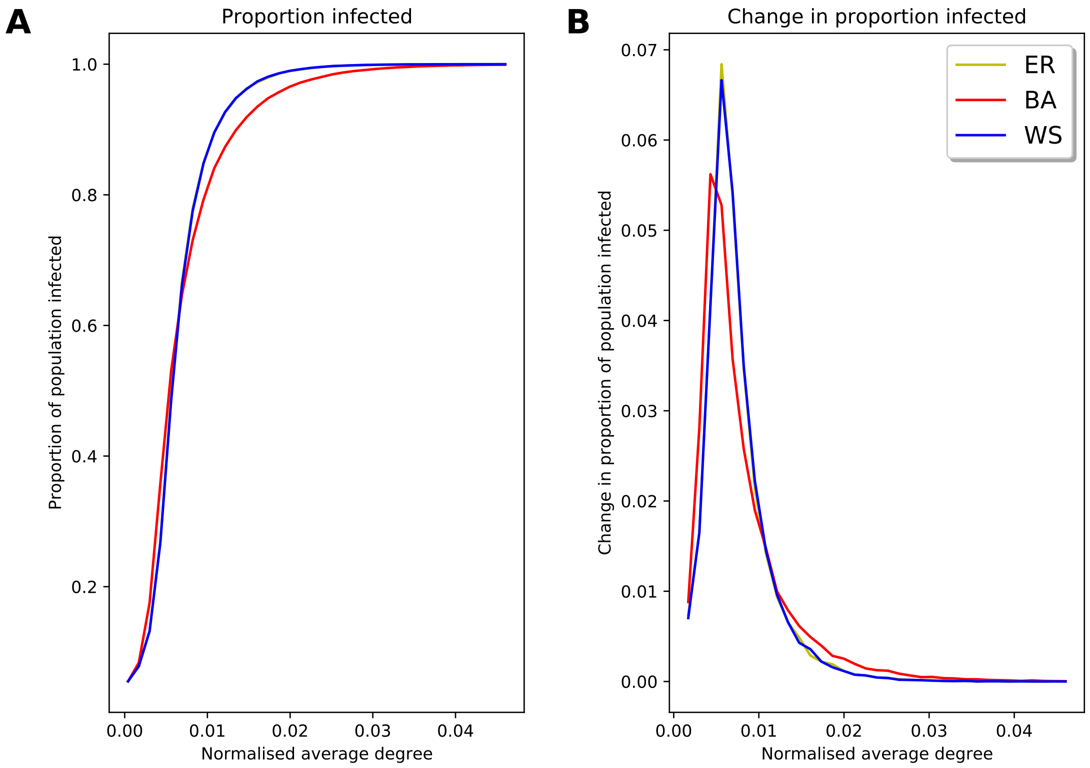

Supplementary Figure 2. Total infection for SIR model on synthetic networks with random edge removal. Proportion of population becoming infected for varying average node degree (A); change in proportion of population becoming infected for varying average node degree (B)

In Supplementary Figure 3 we look at the peak infection rates and the times to stability and peak infection, similarly to Figure 7. Once again, the general trend in the random edge removal case mirrors the one seen in random node removal. The peak infection level decreases as one removes edges, and the rate of this decrease increases as edges are removed. The times to the peak also increase as one removes edges, before decreasing in the extreme cases. The WS and ER networks show similar trends, with the BA being more gradual, with lower peaks but larger regions of change.

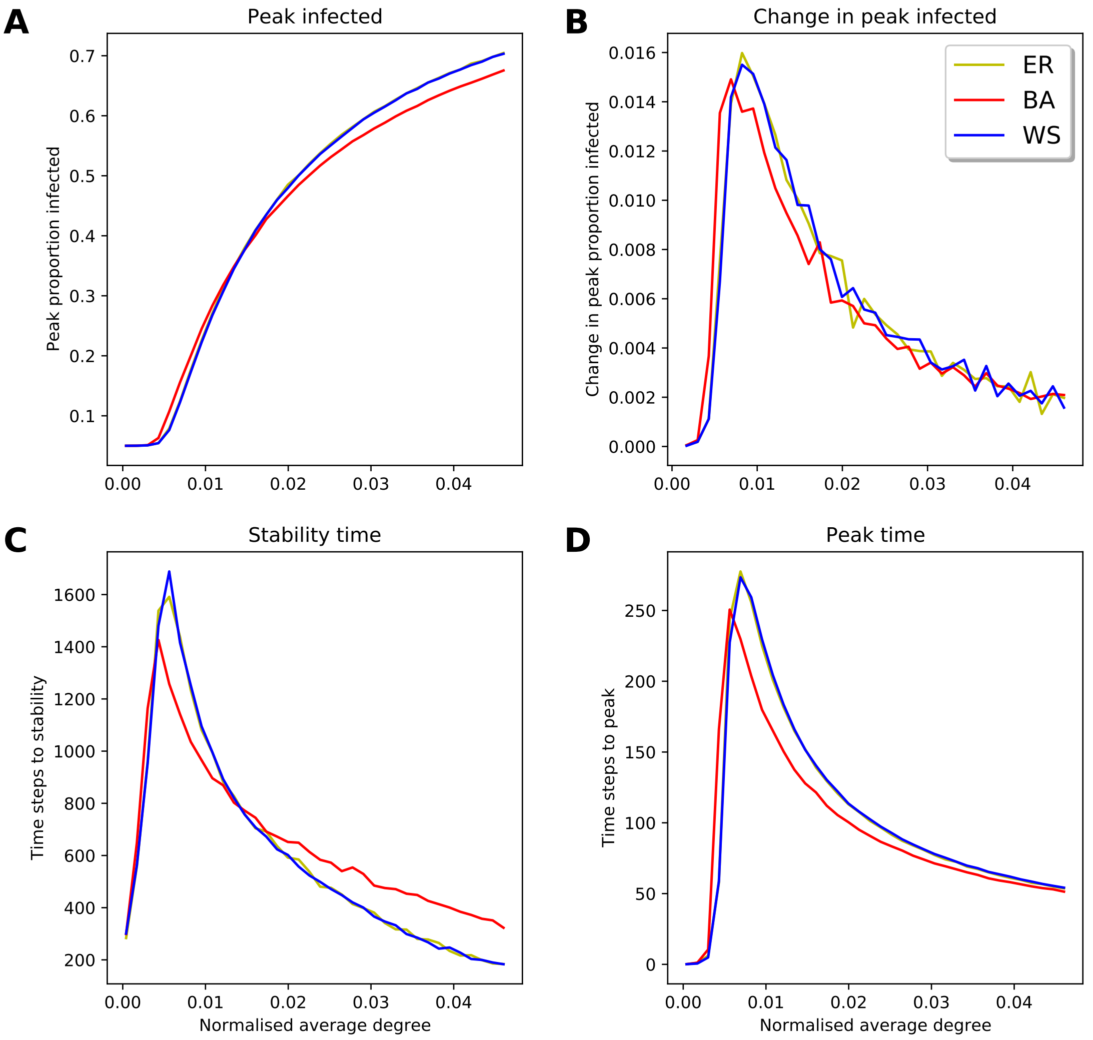

Supplementary Figure 3. Peak infection and stability time for SIR model on synthetic networks with random edge removal. Peak proportion of population infected at same time (A); change in peak proportion of population infected at same time (B); number of time steps to stability (C); number of time steps to peak infection (D)

2.2 Threshold Model

Supplementary Figure 4 replicates Figure 8, showing the proportion of the population becoming infected in the Threshold model as one removes edges. Similarly to the node removal case, initially there is no additional spread; once enough edges have been removed, however, the entire population becomes infected. As before, the ER networks have this change over the smallest region, while the WS networks take place over the largest region.

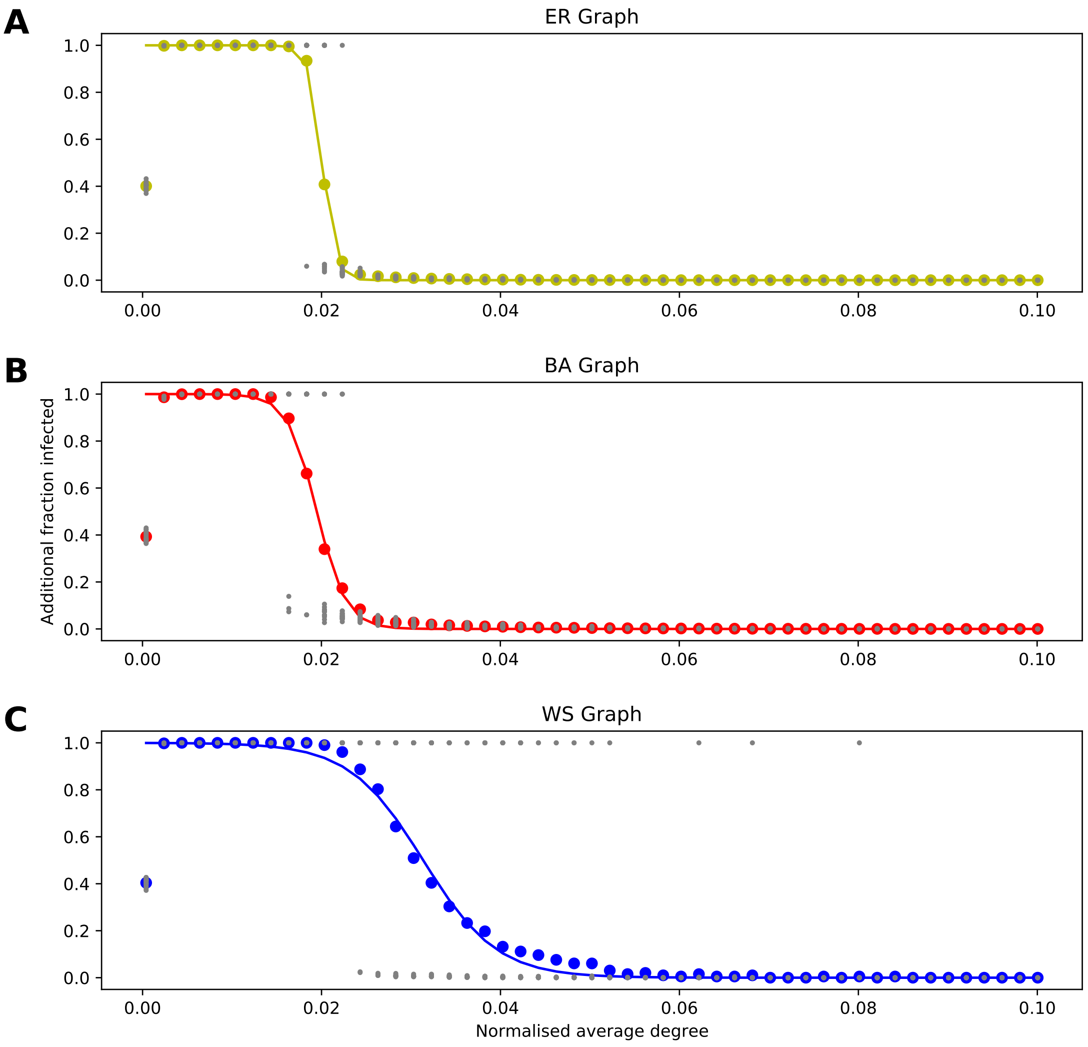

Supplementary Figure 4. Threshold model on synthetic networks with random edge removal. ER network (A); BA network (B); WS network (C)

2.3 Real-World Networks

Supplementary Figure 5 replicates Figure 13, showing how the proportion of the population infected changes as one removes edges. The x-axis is the normalised average degree in the network after the edges have been removed, and the derivatives have been scaled to be relative to 0.0001 and 0.00001 changes in normalised average degree for the *ArXiv* and Slashdot.org networks respectively. Once again, we see the non-linearity of the impact of data handling errors, with similar results as were observed in the node removal on the real-world networks, as well as in the node and edge removal in the synthetic networks.

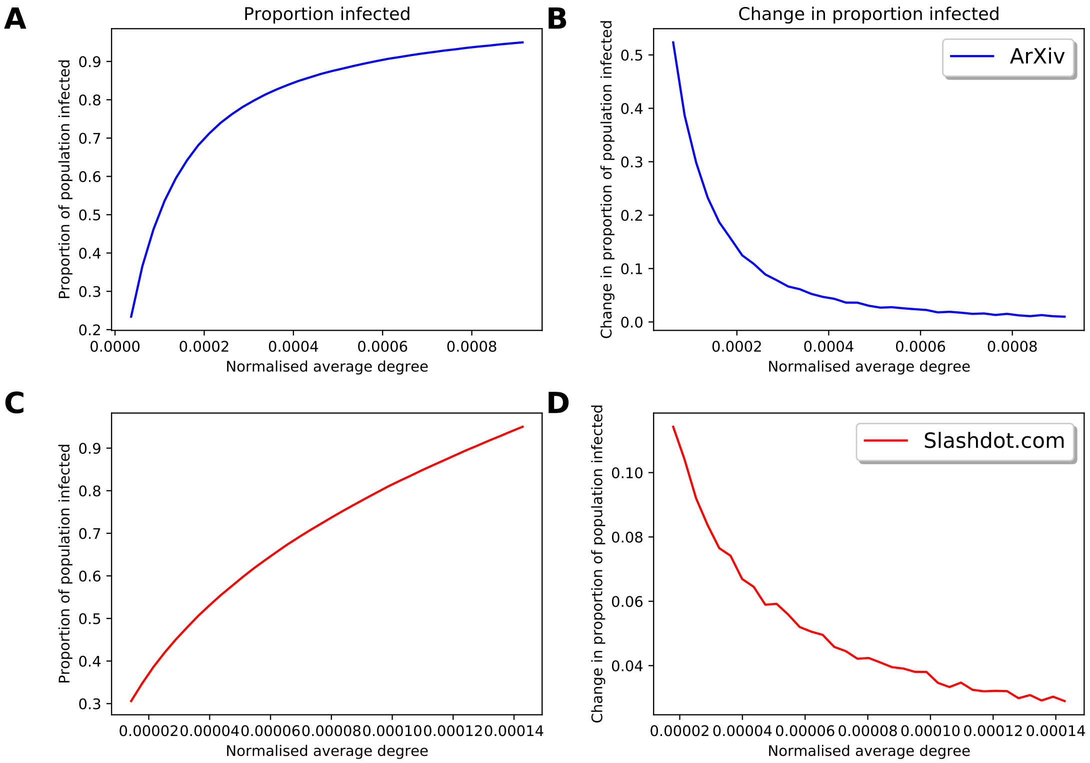

Supplementary Figure 5. Total infection for SIR model on real-world networks with random edge removal. Proportion of population becoming infected for varying average degree on *ArXiv* network and Slashdot.org network (A and C respectively); change in proportion of population becoming infected for varying average degree on *ArXiv* network and Slashdot.org network (B and D respectively)

3 THRESHOLD MODEL ON REAL-WORLD NETWORKS

Supplementary Figure 6 shows the results of running the Threshold model on the real-world networks with the same parameters as were used with the synthetic networks (Figure 8). At first glance, these results seem to differ from those observed in the synthetic networks. We again see non-linear behaviour; now, however, the entire population is initially infected and this infected proportion reduces as nodes are removed. We also see that individual trials no longer only result in full spread or no spread. These differences are most likely a result of the different network sizes and densities. In Figure 8, we note that the curves in the extreme left seem to begin sloping downwards, which could correspond to what is observed in Figure 6.

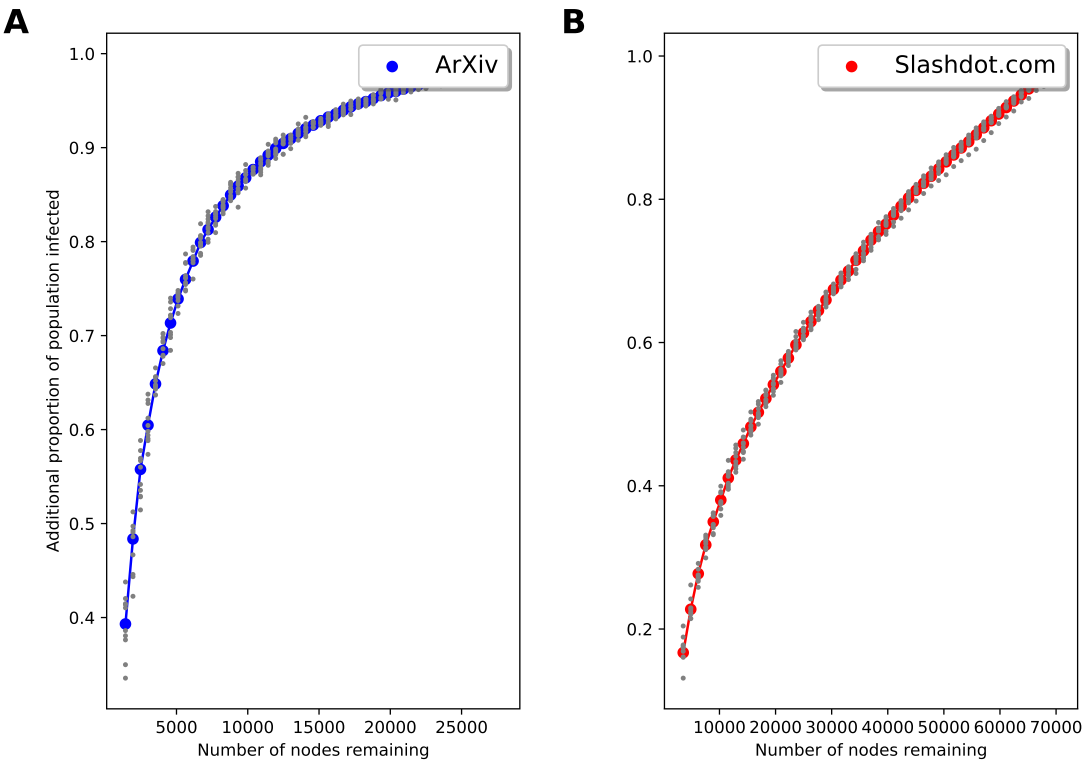

Supplementary Figure 6. Threshold model on real-world networks with random node removal. Additional proportion of nodes infected for varying number of nodes remaining on *ArXiv* network and Slashdot.org network (A and B respectively)

In order to verify this, we run experiments on the synthetic networks removing nodes until only 10 are remaining in each network. Supplementary Figure 7 shows the outcome of the running the model when between 100 and 10 nodes remain in the network, equivalent to a zoomed-in view of the extreme left of Figure 8. Once again, the x-axis represent the number of nodes remaining in the network, and the y-axis the additional proportion of the becoming infected. These results are similar to what is observed in Supplementary Figure 6, providing evidence that the size and density of a network can vary which regions of data handling impact one falls in. For this model, removing nodes from the dense network can increase the spread of a disease, up to a point where the entire population becomes infected. If nodes are continually removed to a point where the network becomes very small, we see the spread decreasing. Alternatively, in a sparse network, removing nodes can reduce the spread of a disease, as seen in the real-world networks. These insights highlight potential areas of further study, such as: what are the network sizes/densities where the behaviour changes, plus how this can vary between different diffusion models.

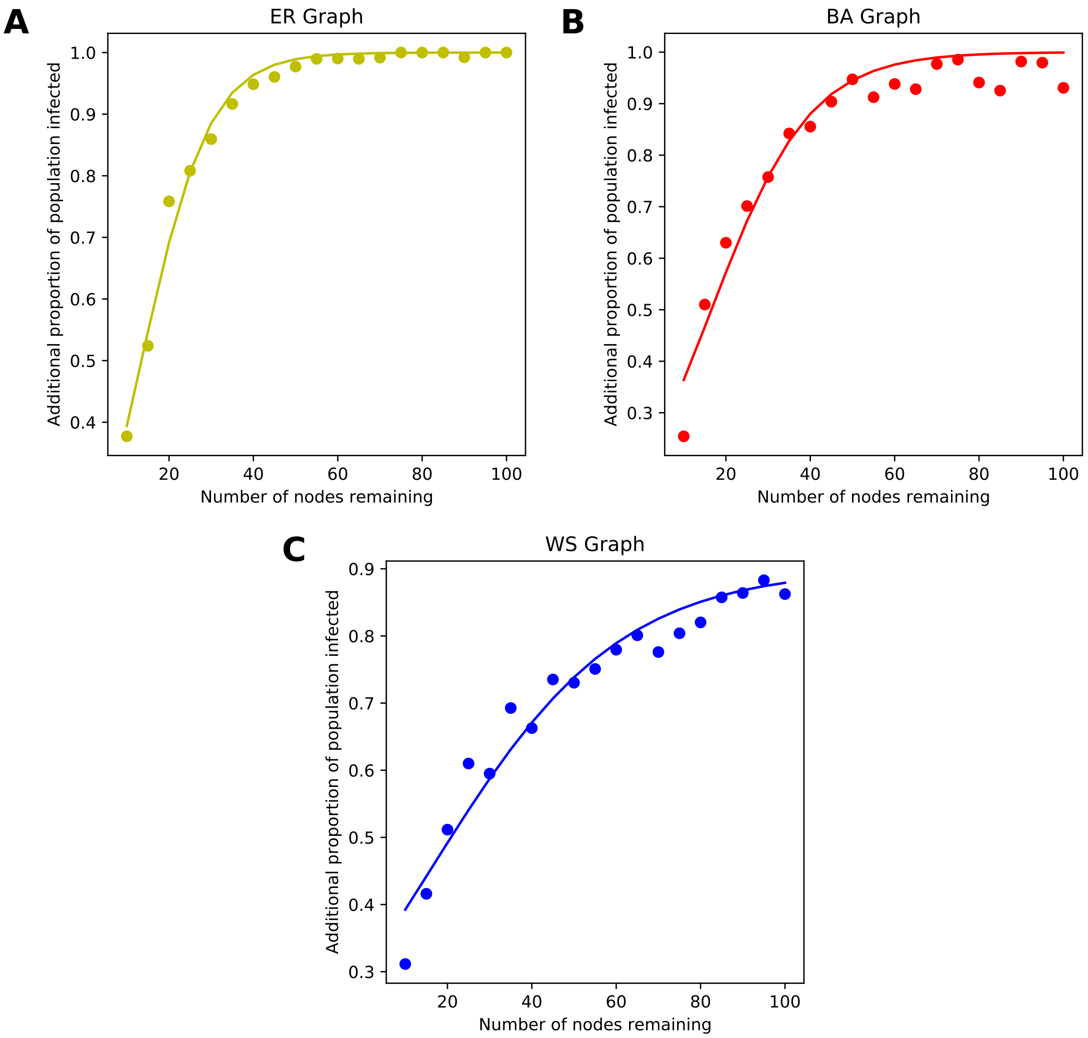

Supplementary Figure 7. Synthetic networks' additional proportion of nodes infected for varying number of nodes remaining. ER, BA, and WS networks (A, B, and C respectively)

4 NODE AGGREGATION ON REAL-WORLD NETWORKS

The node aggregation algorithm applied to the synthetic networks cannot be consistently applied to the real-world networks tested. The real-world networks are assumed to have already been processed through some data handling procedure, for which we do not have the details nor the original raw data. As such, applying the algorithm detailed previously would violate this assumption and result in our having networks that do not reflect real-world networks.

We instead utilise a simple algorithm intended to replicate imperfect data integration. The real-world network is randomly split into two networks with an equal number of edges, intended to represent networks collected from different data sources. These two networks are then integrated, where equivalent nodes appearing in both networks are correctly combined into one node with some accuracy. This is similar in practice to randomly disaggregating nodes by splitting them into two and randomly dividing the edges between them. Algorithm 1 describes the algorithm implemented.

Supplementary Figure 8 shows the result of applying this node aggregation algorithm to the real-world networks and then running the SIR model on them with the parameters used in section 5.3. We see that lowering the accuracy of the node aggregation algorithm leads to a linear increase in the number of node disaggregations, and a linear decrease in the proportion of the population becoming infected. The change in the proportion of the population infected is small – from 0.95 to approximately 0.91. This example illustrates that different data handling methods may not suffer from the non-linearity observed in other

Algorithm 1 Real-world network aggregation simulation

Set aggregation accuracy, α
Divide edge set, E, randomly in half into E_1 and E_2
Define *intersection_nodes* = intersection of nodes in E_1 and E_2
for node i in *intersection_nodes* **do**
    Generate random uniform random variable U between 0 and 1
    if $U > \alpha$ **then**
        Relabel node i in all edges in E_2 to node i_{adj}
    end if
end for
Combine E_1 and E_2 to create one network

cases. Here, the model is fairly robust in both networks to node disaggregation when considering the high-level metric of population infected.

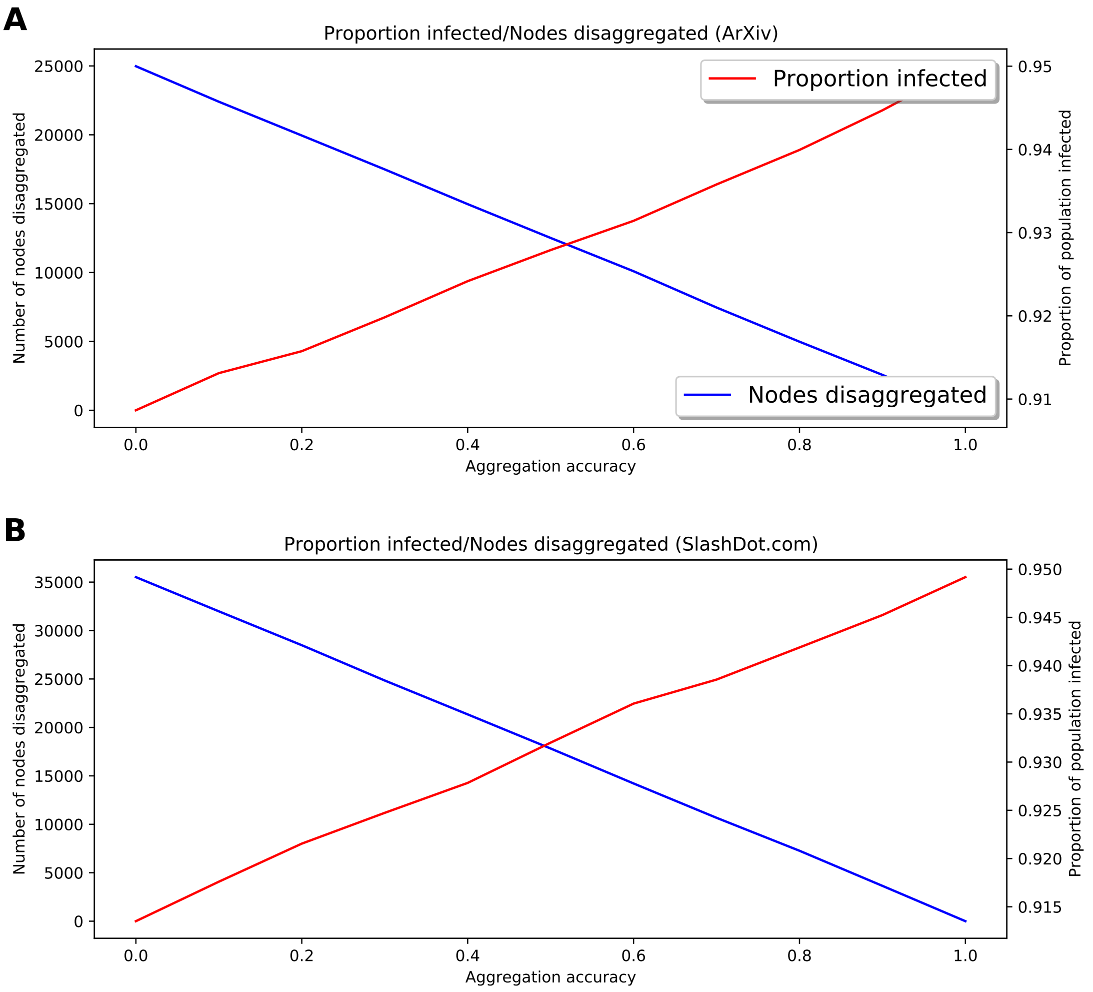

Supplementary Figure 8. Node aggregation and the SIR model on real-world networks. *ArXiv* network and Slashdot.org network (A and B respectively)
